# HrpA anchors meningococci to the dynein motor and affects the balance between apoptosis and pyroptosis

**DOI:** 10.1186/s12929-022-00829-8

**Published:** 2022-06-28

**Authors:** Adelfia Talà, Flora Guerra, Matteo Calcagnile, Roberta Romano, Silvia Caterina Resta, Aurora Paiano, Mario Chiariello, Graziano Pizzolante, Cecilia Bucci, Pietro Alifano

**Affiliations:** 1 Department of Biological and Environmental Sciences and Technologies (DiSTeBA), University of Salento, Via Provinciale Monteroni n. 165, 73100 Lecce, Italy; 2 Core Research Laboratory-Siena, Institute for Cancer Research and Prevention (ISPRO), 53100 Siena, Italy; 3 Institute of Clinical Physiology (IFC), National Research Council (CNR), 53100 Siena, Italy

**Keywords:** Host–pathogen interaction, Dynein, Apoptosis, Pyroptosis, Cell death, Bacteria, Two-hybrid system

## Abstract

**Background:**

In *Neisseria meningitidis* the HrpA/HrpB two-partner secretion system (TPS) was implicated in diverse functions including meningococcal competition, biofilm formation, adherence to epithelial cells, intracellular survival and vacuolar escape. These diverse functions could be attributed to distinct domains of secreted HrpA.

**Methods:**

A yeast two-hybrid screening, in vitro pull-down assay and immunofluorescence microscopy experiments were used to investigate the interaction between HrpA and the dynein light-chain, Tctex-type 1 (DYNLT1). In silico modeling was used to analyze HrpA structure. Western blot analysis was used to investigate apoptotic and pyroptotic markers.

**Results:**

The HrpA carboxy-terminal region acts as a manganese-dependent cell lysin, while the results of a yeast two-hybrid screening demonstrated that the HrpA middle region has the ability to bind the dynein light-chain, Tctex-type 1 (DYNLT1). This interaction was confirmed by in vitro pull-down assay and immunofluorescence microscopy experiments showing co-localization of *N. meningitidis* with DYNLT1 in infected epithelial cells. In silico modeling revealed that the HrpA-M interface interacting with the DYNLT1 has similarity with capsid proteins of neurotropic viruses that interact with the DYNLT1. Indeed, we found that HrpA plays a key role in infection of and meningococcal trafficking within neuronal cells, and is implicated in the modulation of the balance between apoptosis and pyroptosis.

**Conclusions:**

Our findings revealed that *N. meningitidis* is able to effectively infect and survive in neuronal cells, and that this ability is dependent on HrpA, which establishes a direct protein–protein interaction with DYNLTI in these cells, suggesting that the HrpA interaction with dynein could be fundamental for *N. meningitidis* spreading inside the neurons. Moreover, we found that the balance between apoptotic and pyroptotic pathways is heavily affected by HrpA.

**Supplementary Information:**

The online version contains supplementary material available at 10.1186/s12929-022-00829-8.

## Background

*Neisseria meningitidis* is a leading cause of two devastating human diseases: meningitis and septicemia. The only known natural reservoir of this pathogen is the human nasopharynx, where it normally resides as a harmless transitory in up to 18% of healthy subjects. In some individuals this common transitory colonizer is able to breach the mucosal barrier, get into bloodstream resulting in septicemia and/or septicemic shock; sometimes it may also cross the blood–brain barrier to reach the subarachnoid space of the leptomeninges causing meningitis with or without septicemia. Both host and bacteria factors seem to be involved in the switch from harmless transitory colonization to devastating disease [1].

Many investigators have attempted to define the interaction between *N. meningitidis* and its host at the molecular and cellular level, and a number of virulence determinants including capsular polysaccharide, lipopolysaccharide, type IV pili, IgA1 protease, surface-adhesive proteins and iron-scavenging systems have been characterized by using either cell and organ culture systems or animal models [2]. In recent years it has become increasingly apparent the importance of secretion systems in the evolution of meningococcal pathogenesis, and this fact is tremendously attractive because the secreted proteins may constitute suitable components of vaccines or targets of therapeutic intervention [3]. Despite the large heterogeneity of secretion systems present in other Gram-negative bacteria, *N. meningitidis* has been shown to use only three secretion pathways: type I, autotransporter (type Va) and two-partner secretion (TPS) (type Vb) systems [3].

TPS is a secretion pathway that is devoted to secretion of large proteins that in many Gram-negative bacteria appear to play key roles in microbe-host interactions, microbial virulence and intraspecific variation, competition and evolution. This secretion pathway includes an exoprotein (generally referred to as TpsA) with an N-proximal module called the “secretion domain” and a channel-forming β-barrel activator/transporter protein (TpsB) that is thought to transport the exoprotein across the outer membrane [4–7]. The best characterized TpsA family members are the filamentous hemagglutinin (FHA) of *Bordetella pertussis* and the high-molecular-weight proteins of *Haemophilus influenzae*, the calcium-independent hemolysins ShlA and HpmA of *Serratia marcescens* and *Proteus mirabilis*, respectively, and CdiA, a protein involved in contact-dependent growth inhibition in *Escherichia coli* [4, 7].

In the meningococcal genome, cluster analysis of TpsA and TpsB proteins revealed the presence of up to three different TPS systems, and some of these systems may contain more than one *tpsA* gene [8]. For instance, in the genome sequence of the reference serogroup B strain MC58, five different *tpsA* genes were identified, two belonging to system 1 (*tpsA1a* and *tpsA1b*), two to system 2 (*tpsA2a* and *tpsA2b*), and one to system 3 (*tpsA3*). In contrast, the genomes of the reference strains FAM18 (serogroup C), 053442 (serogroup C) and Z2491 (serogroup A) contain only a single *tpsA* gene belonging to system 1. System 1 appears to be meningococcus-specific, while systems 2, and 3 are overrepresented in disease isolates compared to carriage isolates, but are present also in *Neisseria lactamica* together with a distinct system 4 that is absent in meningococci [8].

System 1 *tpsB* and *tpsA* genes are located in specific genetic islands. Downstream of the *tpsA* genes, several 5’-end-truncated *tpsA*-related open reading frames (ORFs) are located, generically referred to as *tpsC* cassettes [8], interspersed with small intervening ORFs (IORFs). The *tpsC* cassettes share sequence similarity with the central region of *tpsA* but show an entirely different 3’-terminal sequence. It has been shown that the system 1 TPS of *N. meningitidis* functions as a toxin-antitoxin fratricide system that inhibits growth of other meningococci competing for the same niche in human host [9]. TpsA1 proteins of *N. meningitidis* mediate growth inhibition, while the downstream ORFs confer immunity to the producing strain. Similarly systems have been described in TpsA (CdiA) proteins of other Gram-negative bacteria, in which contact-dependent inhibitory activity resides within the carboxy-terminal region of CdiA (CdiA-CT) downstream of VENN peptide motif, and CdiI anti-toxin binds and inactivates cognate CdiA-CT, but not heterologous CdiA-CT [7]. Low-frequency recombination with silent *tpsC* cassettes may introduce different toxic modules at the variable C terminus of meningococcal TpsA [9].

Apart from its involvement in the intricate immunity network that may control the high diversity of meningococcal populations in Nature, TpsA1 proteins of *N. meningitidis* (referred to as HrpA for “hemagglutinin/hemolysin-related proteins A” in previous studies and herein) have reported roles in host–pathogen interaction. It was demonstrated that deletion of *hrpB* (*tpsB1*) in an unencapsulated and LPS-truncated strain led to significant decrease in adherence to epithelial cells [10]. Also the HrpA protein is proteolytically processed to a ca. 180 KDa form and secreted in an HrpB-dependent process, while a small proportion of HrpA remains associated with the bacterial cell and contributes to its interaction with epithelial cells [10]. Our research group showed that HrpA proteins play a key role in intracellular survival by mediating the bacterial escape from the internalization vacuole into the cytoplasm and exit from infected epithelial cells, and that these proteins appear to act as manganese-dependent cell lysins [11]. The importance of HrpA in biofilm formation on human bronchial epithelial (HBE) cells in both encapsulated and unencapsulated strains of *N. meningitidis* was also demonstrated [12]. These studies also established that *hrpB* and *hrpA* genes are up-regulated upon contact with host cells, in the intracellular environment of host cell, and in situations of anaerobiosis [11, 12].

As the multiple roles of HrpA proteins may be related to multiple domains for interaction with cellular or bacterial ligands we have, in the present study, tested this hypothesis by using the yeast two-hybrid system to identify HrpA-interacting proteins in human cells.

## Methods

### Bacterial strains and growth conditions

*N. meningitidis* B1940 [B:NT:P1.3,6,15; LOS immunotype L3,7,9] is piliated and expresses Opa and Opc adhesins [13]. MC58 is an international reference strain [14]. The meningococcal strains were cultured on GC agar base (OXOID) supplemented with 1% (v/v) Polyvitox (OXOID) at 37 °C in a 5% CO_2_ incubator as described [15, 16].

*Escherichia coli* strain DH5α [F^−^ Φ80d *lacZ*ΔM15 *endA1 recA1 hsdR17 supE44 thi*-1 l^−^ *gyrA96* Δ(*lacZYA-argF*) *U169*] was used in cloning procedures. This strain was grown in Luria Bertani (LB) medium. To allow plasmid selection, LB medium was supplemented with ampicillin (75 µg ml^−1^) or kanamycin (50 µg ml^−1^). *E. coli* strain BL21 (DE3) [B F^–^ *ompT gal dcm lon hsdS*_*B*_(*r*_*B*_^–^*m*_*B*_^–^) λ(DE3 [*lacI lacUV5*-*T7p07 ind1 sam7 nin5*]) [*malB*^+^]_K-12_(λ^S^)] was used for expression of His-tagged proteins.

*Saccharomyces cerevisiae* AH109 strain (*MATa*, *trp1-901*, *leu2-3*, *112*, *ura3-52*, *his3-200*, *gal4Δ*, *gal80Δ*, *LYS2::GAL1UAS-GAL1TATA-HIS3*, *GAL2UAS-GAL2TATA-ADE2*, *URA3::MEL1UAS-MEL1TATA-lacZ*, *MEL1*) was used for two-hybrid assay [17, 18]. This strain was cultivated with synthetic dropout medium (SD).

### DNA procedures, plasmids and cloning procedures

**Fig. 1:**
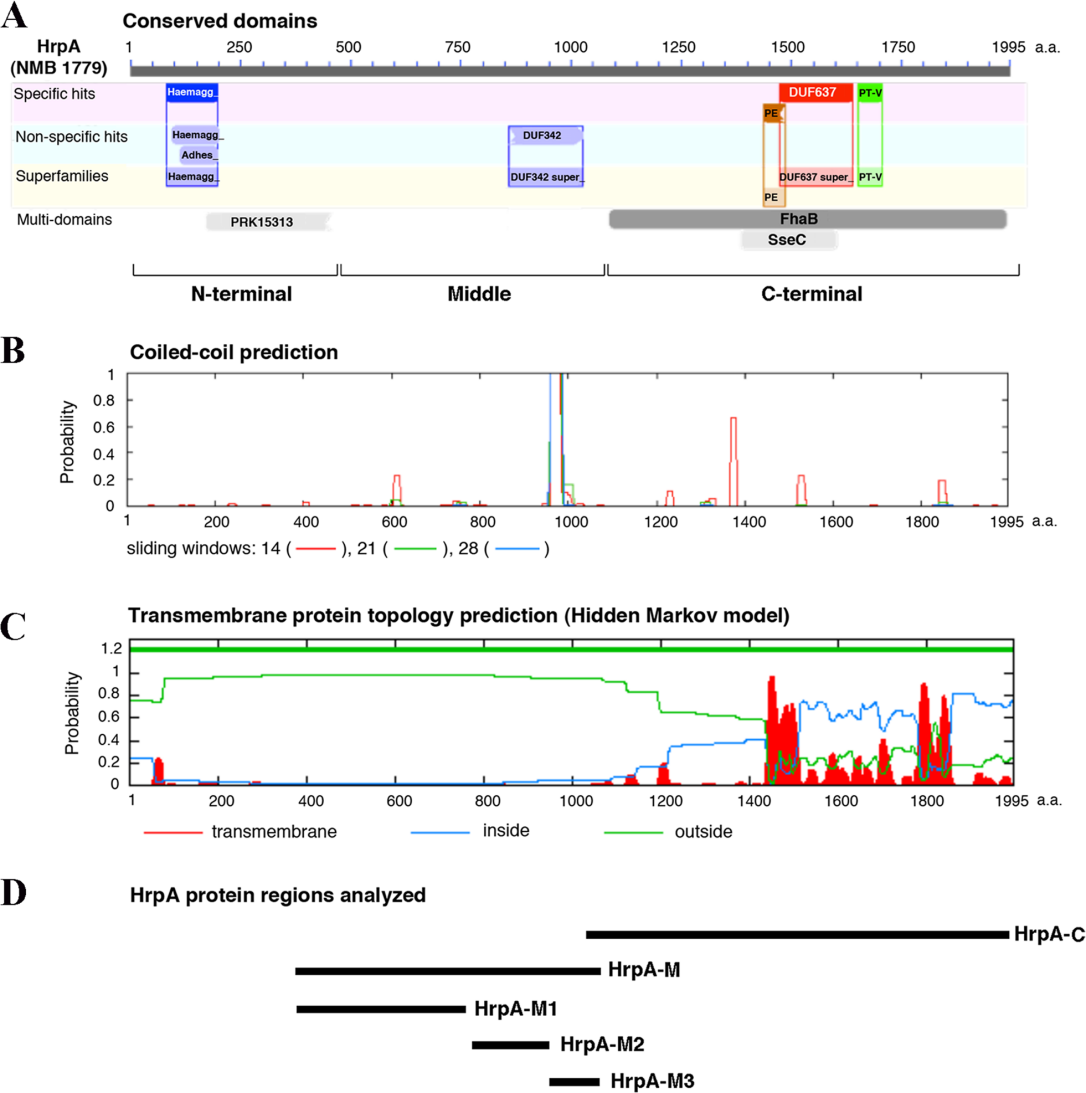
Structural domains of *N. meningitidis* HrpA protein and study design. **A** Conserved domains within the N-terminal, middle and C-terminal regions of HrpA (NMB1779) are depicted. **B**, **C** Coiled coil **B** and transmembrane topology **C** predictions of HrpA are illustrated. **D** HrpA protein regions that were analyzed in this study are shown

Genomic DNA from *N. meningitidis* was prepared as described [19]. Oligonucleotides used as primers in PCR reactions are listed in Additional file 1: Table S1. The amplification reactions generally consisted of 35 cycles including 45 s of denaturation at 94 °C, 45 s of annealing at 55 °C and 45 s of extension at 72 °C. The DNA from strain MC58 was used as a template. PCR reactions were used to generate the following amplicons that were used in cloning procedures and are shown in Fig. 1d: HrpA-C (primer pair: F_3_/R_3_), 2763 bp long amplicon (from nt 3100 to nt 5862 of NMB1779, corresponding to a.a. 1035–1995); HrpA-M (primer pair: F_2_/R_2_): 2072 bp long amplicon (from nt 1129 to nt 3200 of NMB1779, corresponding to a.a. 377–1066); HrpA-M1 (primer pair: F_2_/R_21_): 1162 bp long amplicon (from nt 1129 to nt 2290 of NMB1779, corresponding to a.a. 377–763); HrpA-M2 (primer pair: F_22_/R_22_): 537 bp long amplicon (from nt 2311 to nt 2847 of NMB1779, corresponding to a.a. 771–949); HrpA-M3 (primer pair: F_23_/R_2_): 326 bp long amplicon (from nt 2875 to nt 3200 of NMB1779, corresponding to a.a. 959–1066).

pET-HrpAC, pET-HrpAM, pET-HrpAM1, pET-HrpAM2, and pET-HrpAM3 were obtained by inserting, respectively, the *NdeI*/*BamHI* restricted HrpA-C, HrpA-M, HrpA-M1, HrpA-M2, and HrpA-M3 amplicons (Fig. 1d) into *NdeI*/*BamHI* restricted vector plasmid pET-16b.

pGBKT7-HrpAM, pGBKT7-HrpAM1, pGBKT7-HrpAM2, and pGBKT7-HrpAM3 were constructed by cloning, respectively, the *NdeI*/*BamHI* restricted HrpA-M, HrpA-M1, HrpA-M2, and HrpA-M3 amplicons (Fig. 1d) into *NdeI*/*BamHI* restricted vector plasmid pGBKT7.

Oligonucleotide synthesis and DNA sequencing were carried by Eurofins Genomics (Germany) by using the cycle sequencing technology (dideoxy chain termination / cycle sequencing) on ABI 3730XL sequencing machines.

### Yeast two-hybrid assay

The construct pGBKT7-HrpAM was used to screen a HeLa cell line cDNA library cloned in the pGADGH vector and a human fetal liver cDNA library cloned in the pACT-2 vector, using AH109 yeast strain as a host [17, 18, 20, 21]. Transformants were plated onto SD medium lacking histidine, leucine and tryptophan. Colonies were picked 5 days later, and then assayed for growth on medium lacking adenine, histidine, leucine and tryptophan for more stringent selection. Specificity tests were performed by co-transforming AH109 yeast strain with pGBKT7-HrpAM, pGBKT7-HrpAM1, pGBKT7-HrpAM2, pGBKT7-HrpAM3, or pGBKT7 and with either the empty vectors (pGADGH or pACT-2) used as a negative control, or the recombinant plasmids (pGADGH-DYNLT1, pGADGH-USO1, pGADGH-FLNB, pGADGH-UBC9, pGADGH-ITIH3 or pACT2-FGA) that were isolated from the initial two-hybrid screening. As a positive control, yeast cells were co-transformed with pGBKT7-Rab7Q67LΔC and pGADGH-RILPC33 [22].

### HrpA-M and HrpA-C protein expression and purification

DNA fragments encoding HrpA-M, HrpA-M2, HrpA-M3, and HrpA-C proteins were introduced in the pET-16b plasmid and were expressed in *E. coli* BL21. In details, transformed bacterial cells were grown at 37 °C until OD_600_ was ~ 0.6, and then gene expression was induced by adding 0.1 mM IPTG for 1 h at 37 °C. Cells were then lysed in 64 mM Tris–HCl pH 8.5, 8 mM MgCl_2_, 2 mM EDTA, 20 mM β-mercaptoethanol, 10 µg/mL lysozyme, 1% sodium deoxycholate, 2 mM PMSF for 1 h at 4 °C with gentle agitation. Lysates were centrifuged at 15,000*g* for 1 h at 4 °C and the supernatant, representing the cytosolic fraction, was used for protein purification. His-tagged proteins were purified incubating cytosolic fractions with Ni–NTA (Nickel-Nitrilotriacetic Acid) resin for 1 h at 4 °C with gentle agitation. The resin was then washed twice with a washing buffer (NaH_2_PO_4_ 50 mM, NaCl 300 mM, Imidazole 20 mM, pH 8.0) and His-tagged proteins were eluted with the elution buffer (NaH_2_PO_4_ 50 mM, NaCl 300 mM, Imidazole 250 mM, pH 8.0).

### Pull-down assay

Pull-down experiments were carried out as described [23, 24]. Briefly, purified His-tagged HrpA-M2 or HrpA-M3 peptides were bound to Ni–NTA resin for 1 h at 4 °C. After two washes in washing buffer (NaH_2_PO_4_ 50 mM, NaCl 300 mM, Imidazole 20 mM, pH 8.0), HeLa cell lysate containing over-expressed Myc-tagged DYNLT1 peptide was added and incubated for 2 h at 4 °C. After three washes with washing buffer, peptides or peptide complexes were eluted with elution buffer (NaH_2_PO_4_ 50 mM, NaCl 300 mM, Imidazole 250 mM, pH 8.0), and processed for western blot analysis. To this purpose, samples were loaded onto SDS-PAGE gels, and separated proteins were transferred onto polyvinylidene fluoride (PVDF) membrane (Millipore, Billerica, MA). The membrane was blocked by 5% milk in PBS for 30 min at room temperature, incubated with the appropriate primary antibody, and then with a goat anti-mouse secondary antibody conjugated to HRP (Biorad 172–1011) (diluted 1:5000). Mouse anti-polyHistidine (H1029 Sigma) or anti-Myc (sc-40, Santa Cruz) were used as primary antibodies. After washing, bands were visualized by using Western blotting luminol reagent (Santa Cruz).

### Cell culture, infection and confocal immunofluorescence microscopy

Infection experiments were carried out with HeLa cells (ATCC Number CCL-2) as previously described [11, 25]. HeLa cells were grown at 37 °C in a 5% CO_2_ incubator in DMEM supplemented with inactivated 10% fetal bovine serum (FBS), 2 mM L-glutamine, penicillin (50 U ml^−1^) and streptomycin (50 μg ml^−1^). Infection experiments were performed as previously described [11, 25–27]. In brief, HeLa cells were infected with *N. meningitidis* strain B1940 at a multiplicity of infection (MOI) of 50 for 1 h. To start the infection bacteria were centrifuged (600 × g) onto cells. Cells were washed twice with phosphate-buffered saline (PBS) to eliminate the majority of extracellular bacteria and exposed to gentamicin to kill remaining extracellular bacteria. Cells were then washed extensively with PBS to remove gentamicin and dead extracellular bacteria. Gentamicin treatment was performed at 100 mg ml^−1^, a concentration tenfold above the minimal inhibitory concentration (MIC) for 30 min. After 6 h, cells were fixed with 3% paraformaldehyde and then permeabilized with saponin incubated with primary antibodies to stain bacteria or intracellular markers and then incubated with secondary antibodies. Primary antibodies were: rabbit polyclonal anti-*N. meningitidis* antibody from ViroStat (catalog number 6121); rabbit polyclonal anti-Tctex1 (H-60) antibody, directed against amino acid 1–60 mapping at the N-terminal of Tctex1 (DYNLT1) of human origin, from Santa Cruz Biotechnology, Inc. (catalog number sc28537); mouse monoclonal anti-DYNLT1 antibody from Sigma (catalog number D9944). Secondary antibodies were: Alexa Fluor 488 rabbit (catalog number A11034, Thermo Fisher), Alexa 488 mouse (catalog number A11029, Thermo Fisher), Alexa 568 rabbit (catalog number A11036, Thermo Fisher). To discriminate intracellular from extracellular bacteria, the fixed cells were incubated with an anti-*N. meningitidis* antibody and then with a Cy5-conjugated secondary antibody to stain extracellular bacteria; subsequently, cells were permeabilized with 0.25% saponin and then incubated with a primary anti-*N. meningitidis* antibody and an Alexa568-conjugated secondary antibodies to stain intracellular and extracellular bacteria. Thus, intracellular bacteria will be red while extracellular will be purple (blue + red).

Mounted specimens were viewed with the ZEISS LSM700 confocal laser-scanning microscope with a 63 × /1.40 NA oil immersion objective. Adobe Photoshop CC 2018 was used to process the images. NSC34 hybrid cell line was also used in infection experiments. NSC34 cells were grown at 37 °C in a 5% CO_2_ incubator in DMEM supplemented with inactivated 10% fetal bovine serum (FBS), 2 mM L-glutamine, penicillin (50 U ml^−1^) and streptomycin (50 μg ml^−1^). To induce differentiation, NSC34 cells were seeded at low density and grown for 48 h. The infection experiments with *N. meningitidis* strains were conducted as described for HeLa cells with the difference that washes with PBS were done with extreme care to avoid the detachment of cells. To discriminate intracellular from extracellular bacteria, the fixed cells were incubated with anti-*N. meningitidis* antibody and then with secondary antibody to stain extracellular bacteria; subsequently, cells were permeabilized with 0.25% saponin and then incubated with primary and secondary antibodies to stain intracellular bacteria.

Finally, HBMEC cells [28] were grown at 37 °C in a 5% CO_2_ incubator in EBM-2 supplemented with Single-Quots (Lonza, Morrisville, USA), and 10% FBS.

Where indicated, cells were treated with Z-VAD (Vinci-Biochem, Florence, Italy), an inhibitor for caspases at the concentration of 25 µM for 24 h.

### Gentamicin protection assay

Gentamicin protection experiments were performed as previously described [11]. Briefly, NSC-34 cells were infected with wild type or *hrpA*-defective mutant strains at a multiplicity of infection (MOI) of 50. Bacteria were centrifuged (600xg) onto cells to start the infection. After 1 h of incubation at 37 °C in a 5% CO_2_ incubator, cells were washed twice with PBS, to eliminate the majority of extracellular bacteria, and exposed to gentamicin 100 µg mL^−1^ (a concentration about tenfold above the minimal inhibitory concentration) for 30 min at 37 °C in a 5% CO_2_ incubator to kill the remaining extracellular bacteria. Intracellular bacteria were protected from gentamicin that is unable to penetrate the eukaryotic cells and kill bacteria located inside the cells. Then, cells were washed extensively with PBS to remove gentamicin and dead extracellular bacteria and re-incubated in fresh culture medium. For quantification of survival/growth of extracellular bacteria, medium was collected at 0, 3, 5, 7 h after infection, serially plated onto GC agar plates, and colony forming units (cfu) were counted the day after. For quantification of intracellular survival/growth of wild type or *hrpA*-defective mutant strains, NSC34 cells were lysed with 0.1% saponin to release intracellular bacteria. Lysates were then serially plated onto GC agar plates to determine the cfu.

### RNA interference and transfection

RNA interference was used to silence DYNLT1 and Dynlt1b in human HeLa and mouse NSC34 cell lines, respectively. Both cell lines were silenced as previously described [29] using Metafectene Si (Biontex, München, Germany) transfection reagent following the manufacturers instructions. After transfection HeLa were incubated for 24 h and NSC34 cells for 48 h before infection and subsequent analyses. Cell lines were transfected with control RNA (scrambled) or with two different predesigned siRNA (Sigma-Aldrich, Eurofins). To perform the rescue experiment, 24 h after silencing HeLa cells were transfected with Myc-tagged DYNLT1 using Metafectene Pro (Biontex, München, Germany) as transfection reagent, and incubated for 24 h before the lysis.

### Western Blotting

After 24 h (for HeLa and NSC34 cells) and 30 h (for HBMEC cells) of infection, cells were lysed in Laemmli Buffer (100 mM Tris–HCl, pH 6.8, containing 4% SDS, 20% glycerol and 0,2% blue bromophenol). The protein amount in cell extracts was quantified loading extracts on Coomassie Blue gel (12% SDS-PAGE) together with a control lysate of known concentration and using ImageJ software to quantify. On each well of SDS-PAGE gels about 20 to 50 μg of lysates were loaded. Western blotting was performed as described [30]. Antibodies against Caspase-8 (Cell Signaling Technology, #9746), Caspase-3 (Cell Signaling Technology, #9665), Caspase-9 (Cell Signaling Technology, #9508), PARP-1 (Cell Signaling Technology, #9532), Dynein (Sigma-aldrich, D9944), Gasdermin E N-terminal (Abcam, ab215191), Caspase-4 (Abcam, ab2381,24), Caspase-11 (Abcam, ab180673), Caspase-1 (Abcam, ab179515), Gasdermin D (Cell Signaling Technology, #39754), Vinculin (Sigma-Aldrich, V9131), GAPDH (Glycerol-3-Phosphate-Dehydrogenase) (Santa Cruz Biotechnology, sc. 25778) were used. Blots were visualized using Clarity and Clarity Max ECL Western Blotting Substrate (Bio-Rad) and images were acquired by the ChemiDoc MP Imaging System and analyzed by Image Lab™ software version 6.0.1 (Bio-Rad).

### Hemolytic activity assay

Hemolytic activity assays were carried out as previously described [11]. Briefly, 10 mL of anticoagulant-treated fresh human whole blood were centrifuged at 2000×*g* for 5 min at room temperature and the resulting plasma fraction was removed from the sample. The pellets were washed 3 times with two volumes of phosphate-buffered saline (PBS) at pH 5.8, mixing by inversion. After the last washing step, a volume of PBS was added, in order to make a 50% (w/v) suspension of human erythrocytes. Purified proteins (about 200 ng) were incubated with human erythrocytes (2% final concentration) either in the absence or in presence of different amounts (0–10 mM) of MgCl_2_, MnCl_2_, CoCl_2_, CuCl_2_, ZnCl_2_, CaCl_2_, FeCl_2_, or NiCl_2_ in final volume of 2 mL. Negative controls were set up in buffer and metal salts, omitting purified proteins. After 90 min of incubation at 37 °C the erythrocytes were removed by centrifugation and the absorbance was determined at 540 nm.

### Bioinformatic analysis and in silico 3D modeling

Multiple sequence alignments of neisserial TpsA proteins were carried out by using the Easy Sequencing in PostScript (ESPript) software [31]. The World Wide Web server of the Pole Bioinformatique Lyonnais (PBIL) – Network Protein Sequence Analysis [32] was used for the analysis of protein secondary structure prediction by DSC method [33]. Coiled-coil prediction was carried out as previously described [34]. Hidden Markov model (TMMOD) was used for transmembrane protein topology prediction [35].

To obtain the models of HrpA (HrpA-M3 and HrpA-C) and human DYLNT1 we used I-Tasser package [36]. This online tool, starting from a sequence, provides five 3D models of a given protein with a score (C-score) to evaluate the quality of the models. I-Tasser modeling starts from protein templates identified by LOMETS [37] in PDB library, and provides a score (Z-score) to identify the best ten templates. The sequences processed by I-Tasser must have a length between 100 and 1000 amino acids. Thus, to obtain the HrpA-C model we submitted on I-Tasser the sequence from the amino acids 981 and 1995. The manganese binding site on this model was predicted by Metal Ion-Binding site prediction and docking server (MIB) [38]. Finally, we used Chimera to refine the figures of the models.

To analyze the interaction between HrpA-M3 and DYNLT1 we generated models of these proteins, and selected the 5jpw PDB crystallographic model of DYNLT1 linked with activin receptor IIB (called DIC2). This last model was modified with Chimera [39] to eliminate liker and DIC2 domains. According to literature, we identified several viral proteins [40] that interact with DYNLT1: UL35 (VP26) of *Human herpesvirus-1* (HHV-1) [41], Viral minor capsid protein L2 of *Human papillomavirus-16* (HPV-16) [42] and cellular CD155 receptor of *Poliovirus* [43]. To validate the docking result of HrpA-M3 *vs* DYNLT1 we used UL35 model (generated by I-Tasser) as a positive docking control. To visualize amino acids in docking sites we used Find Clashes/Contacts of Chimera package [39], which selected the contact amino acids enabling the user to mark them with different colors. The multidomain similarity and homology between HrpA-M3 and viral proteins was obtained by pairwise alignment performed by LALIGN [44]. Docking simulations were carried out by using PatchDock [45], and FireDock [46, 47] to refine the interactions, and to obtain a numerical estimation of binding energy (KJ/mol).

### Statistical analysis

The colocalization rate was determined by Zen 2011 software (Carl Zeiss, Oberkochen, Germany) as the weighted colocalization coefficient of DYLT1 and *N. meningitis* bacteria, as previously described [48]. Measures were obtained by analyzing at least 50 cells/sample and at least 20 cells/sample for at least three different experiments. All experiments were repeated at least three times and the error bars represent the standard error of the mean (S.E.M.). All statistical analyses were performed on data through t-test (*p < 0.05, * p < 0.01, and ***p < 0.001).

## Results

### HrpA structural domains and study design

Multiple sequence alignments of neisserial TpsA proteins with the Easy Sequencing in PostScript (ESPript) software [31] revealed that they are modular and consist of three regions: a conserved N-terminal region of ~ 500 amino acids, a highly conserved middle region of ~ 600 amino-acid residues, and a variable C-terminal region of ~ 900 amino-acid residues (Additional file 1: Fig. S1). In this study we focused on HrpA (TpsA1a, NMB1779 in the reference strain MC58). The N-terminal region of HrpA (HrpA-N) is characterized by the presence of a signal peptide with transmembrane domain (Fig. 1C), an N-terminal hemagglutinin/adhesin domain, and PRK15313 domain related to MisL autotransporter (Fig. 1A). When we began this study, little information was available for the middle region of HrpA (HrpA-M) except for the presence of a conserved protein domain family DUF342 of unknown function (Fig. 1A) with two alpha helices forming a coiled-coil structure at the HrpA-M C-terminus (Fig. 1B). The C-terminal region of HrpA (HrpA-C) spans the conserved filamentous hemagglutinin/hemolysin multi-domain FhaB [49, 50], which, in turn, contains the polymorphic and antigenically variable Pro-Glu (PE) protein domain, implicated in immunostimulation and virulence in mycobacteria [51] and the DUF637 domain that is present in conserved regions of bacterial hemagglutinins/hemolysins.

The HrpA protein domains PE and DUF637 also overlap the secretion system effector C (SseC) family domain [52]. In *Salmonella enterica* SseC, SseB and SseD, are secreted by the Salmonella pathogenicity island 2 (SPI2) type III secretion system (TTSS), and form a complex on the bacterial cell surface required for the translocation of known SPI2 effector proteins such as SseJ. SseB forms a filament connecting the SPI2 TTSS to the phagosomal membrane, allowing insertion of SseC into the phagosomal membrane [53]. The Hidden Markov model predicted extensive transmembrane topology in the SseC-like domain of HrpA (Fig. 1C).

Downstream of the hemagglutinin/hemolysin DUF637 domain, the VENN motif of polymorphic toxins (PT-V) is located (Fig. 1A) followed by a region with extensive transmembrane topology (Fig. 1C). PT-VENN domains are located immediately upstream of the CdiA-CT region. The function of VENN motif is not yet known, but it may play a role in autocleavage to release the CdiA-CT toxin for delivery into target bacteria [7].

These preliminary observations about the protein topology and the structural domains of HrpA guided the rationale of this study that was aimed at testing the hypothesis that the multiple roles of HrpA [9–12] may be related to multiple domains for interaction with cellular or bacterial ligands. To pursue this aim, different regions of the protein corresponding to distinct regions or structural domains were analyzed by using different approaches (Fig. 1D).

### The HrpA-C region possesses Mn^2+^-dependent hemolytic activity

In a previous study, the results of hemolytic tests with human erythrocytes suggested that the secreted HrpA proteins could act as manganese-dependent lysins [11]. Broth culture supernatants from serogroup B strain B1940 exhibited hemolytic activity when assays were carried out in the presence of manganese at pH 5.8. The hemolytic activity was significantly reduced in broth culture supernatants from B1940 ΩhrpA mutant compared with that of the parental B1940 strain, and almost fully restored by genetic complementation [11].

Here we set out to identify the structural domain of HrpA with the hemolytic activity, and we focused our attention on the C-terminal region of HrpA (HrpA-C) harboring the FhaB domain. The DNA region encoding the HrpA-C (Fig. 1D) was cloned in pET-16b vector, and the recombinant N-terminal His-tagged HrpA-C protein was expressed in *E. coli* BL21(DE3) cells transformed with pETHrpA-C. Expression of the recombinant protein in IPTG-induced bacteria was verified by SDS-PAGE (Additional file 1: Fig. S2A). Protein yields were higher at earlier induction times, and most of the His-tagged HrpA-C protein could be detected in the insoluble fraction of the crude extract (S30 pellet) (Additional file 1: Fig. S2B). Lower amounts of protein could be found in the soluble fraction (S30 supernatant) possibly due to proteolytic instability (Additional file 1: Fig. S2B). At variance with the His-tagged HrpA-C protein, the His-tagged HrpA-M protein corresponding to the middle region of HrpA could be detected in the soluble fraction of the crude extract (Additional file 1: Fig. 2SC, D) when it was expressed in *E. coli* BL21(DE3). His-tagged HrpA-C and HrpA-M proteins were then purified from the cytosolic fraction using the Ni–NTA resin (Additional file 1: Fig. S5).

**Fig. 2:**
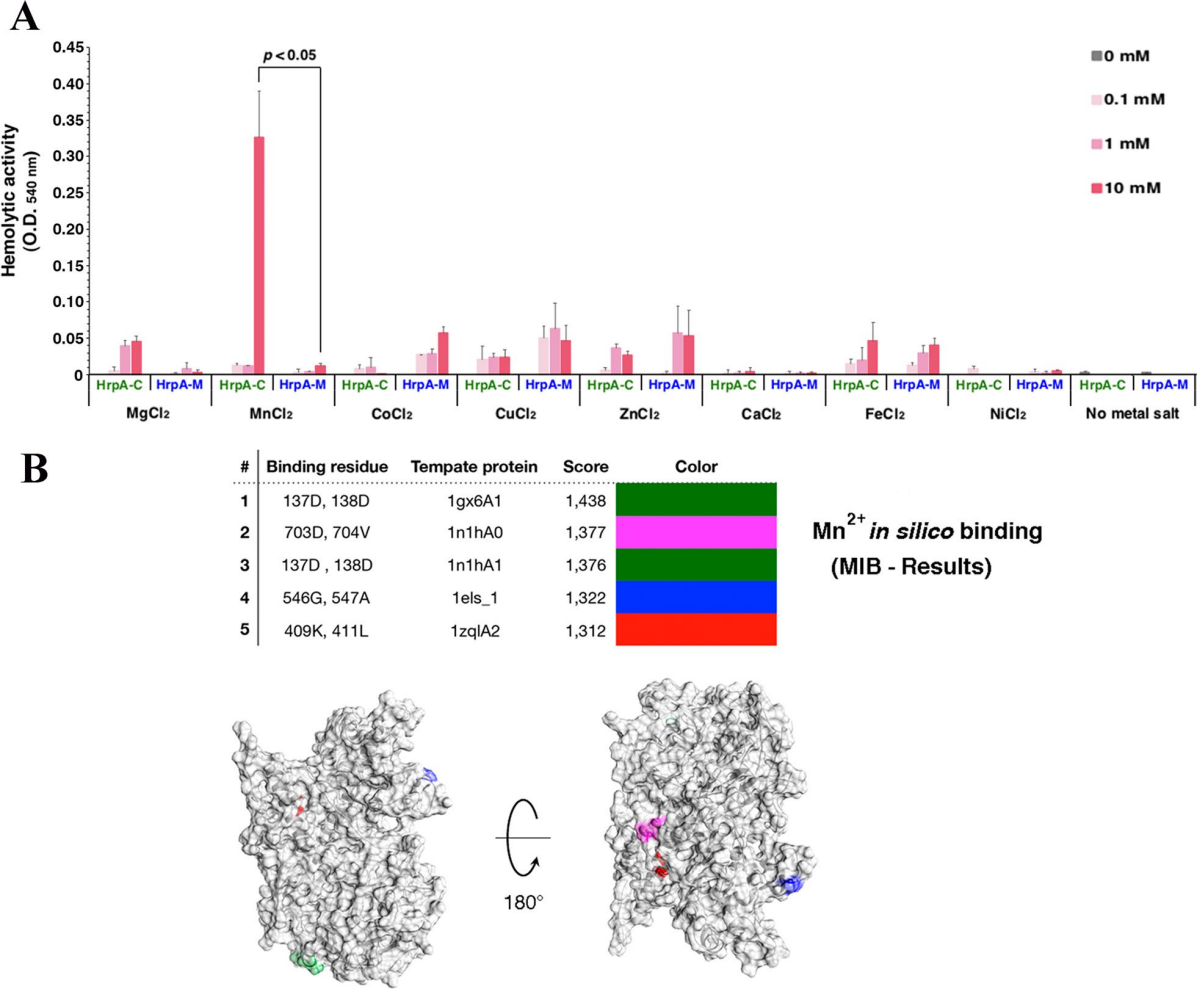
Hemolytic activity of the C-terminal (HrpA-C) and middle (HrpA-M) region of HrpA and in silico interaction between HrpA-C and Mn^2+^ ion. **A** Hemolytic activity of HrpA-C and HrpA-M against human erythrocytes was assayed either in the absence or in the presence of different amounts (0–10 mM) of MgCl_2_, MnCl_2_, CoCl_2_, CuCl_2_, ZnCl_2_, CaCl_2_, FeCl_2_, or NiCl_2_. All data shown in the histogram plot were normalized with respect to their corresponding negative control. The data in the histogram are presented as means ± S.D. of at least three independent experiments done in triplicates. **B** In silico interaction between HrpA-C and Mn^2+^ ion was modeled using the Metal Ion-Binding site prediction and docking server (MIB). Binding residues, template proteins, and score are reported. Putative Mn^2+^ binding sites of HrpA-C were mapped and visualized by Chimera (bottom)

Hemolytic assays were carried out with His-tagged HrpA-C and HrpA-M proteins recovered from the soluble fraction. At moderately acidic pH (pH 5.8) significant (p < 0.05) hemolysis was detected when the His-tagged HrpA-C protein was incubated with the erythrocytes in the presence of 10 mM MnCl2, in comparison with the hemolysis detected when HrpA-M protein was added (Fig. 2A). The hemolytic activity was very weak at 0.1 or 1 mM MnCl2, or in the presence of MgCl_2_, CoCl_2_, CuCl_2_, ZnCl2, FeCl2, in the range 0.1–10 mM, and absent in the presence of CaCl2, and NiCl2, or in the absence of divalent metal salts (Fig. 2A). These data indicate that the hemolytic activity is confined to the C-terminal region of HrpA. Notably, the presence of putative Mn^2+^-binding sites in the C-terminal region of HrpA was predicted by in silico analysis (Fig. 2B).

### HrpA-M region has the ability to interact with host proteins implicated in diverse processes

**Fig. 3:**
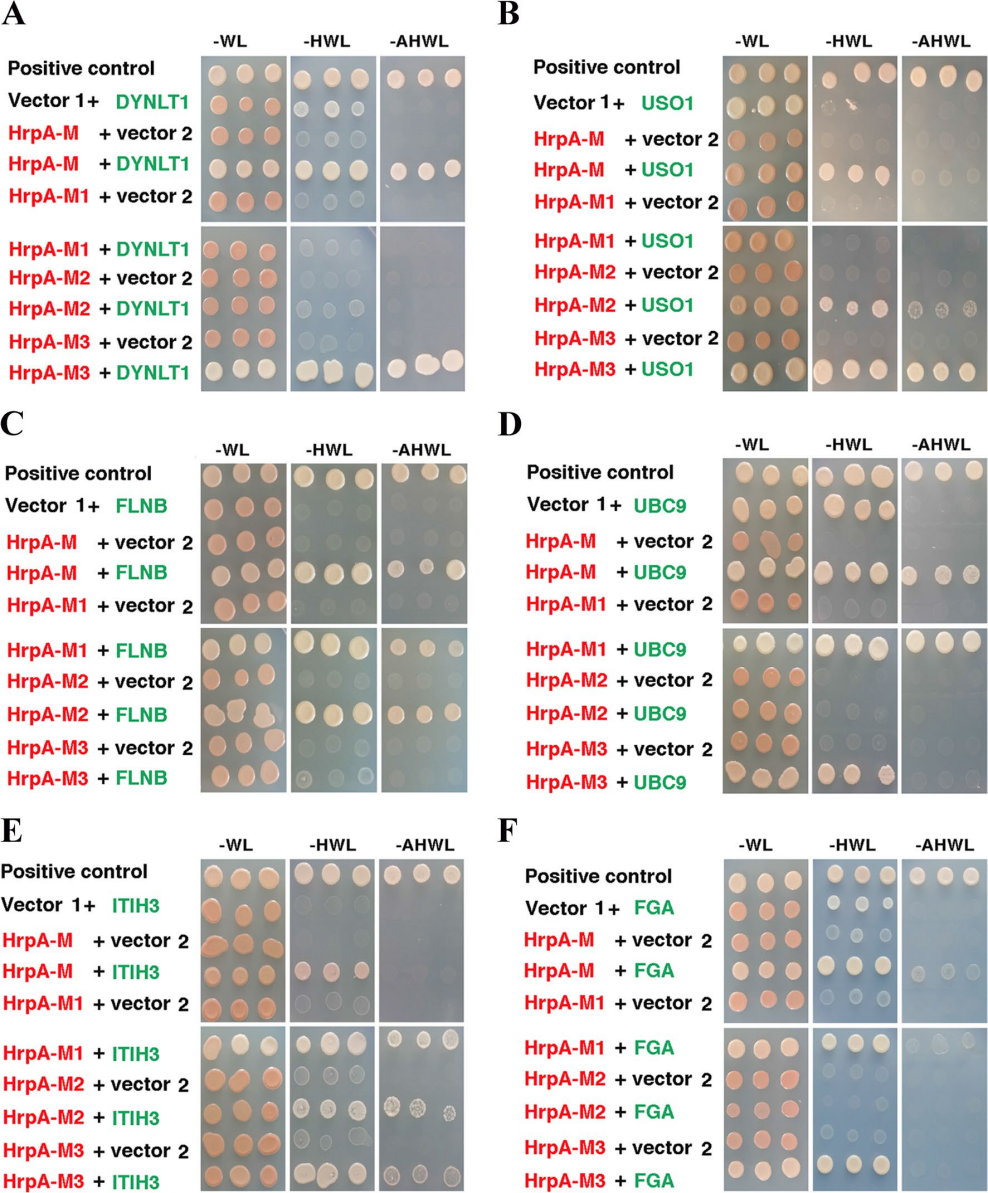
Interaction of host proteins with HrpA-M and sub-regions HrpA-M1, HrpA-M2 and HrpA-M3. Results of yeast two-hybrid tests are shown. In all panels: Vector 1 = pGBKT7; vector 2 = pGADGH or pACT2; HrpA-M = pGBKT7-HrpAM; HrpA-M1 = pGBKT7-HrpAM1; HrpA-M2 = pGBKT7-HrpAM2; HrpA-M3 = pGBKT7-HrpAM3; Positive control = pGBKT7-Rab7Q67LΔC + pGADGH-RILPC33; -WL = medium without tryptophan and leucine; -HWL = medium without histidine, tryptophan and leucine; -AHWL = medium without adenine, histidine, tryptophan and leucine. In **A** DYNLT1 = pGADGH-DYNLT1. In **B** USO1 = pGADGH-USO1. In panel **C**: FLNB = pGADGH-FLNB. In **D** UBC9 = pGADGH-UBC9. In **E** ITIH3 = pGADGH-ITIH3. In **F** FGA = pACT2-FGA

The function of HrpA-M was investigated by yeast two-hybrid screening with the aim to identify putative host interacting proteins. Plasmid pGBKT7-HrpAM expressing a chimeric protein consisting of the N-terminal Gal4 DNA binding domain translationally fused with HrpA-M was used as a “bait” to screen two distinct cDNA libraries, one of HeLa cells and the other of human fetal liver, encoding proteins as C-terminal fusions with the transcriptional activation domain of Gal4, which were used as “preys”. From a total of about 10^7^ primary transformants, 8 transformants from HeLa cell line cDNA library, and 182 from human fetal liver cDNA library survived the initial selection on medium lacking leucine, tryptophan and histidine. Clones were then tested for beta-galactosidase activity, and all clones of the HeLa library and about a quarter of the clones of the fetal liver library were positive. Of these clones 6 from the HeLa library and only 5 from the fetal liver library encoded true positives that did not activate transcription in the presence of a non-specific test bait (data not shown). Also, these clones did not activate transcription in the absence of the HrpA-M bait and some of them survived more stringent selection on medium lacking leucine, tryptophan, histidine and adenine (Fig. 3).

Sequence analysis of the DNA clones from the positive transformants revealed that several putative interactors were fished twice within the same library, including the SUMO-conjugating enzyme (UBC9) and the general vesicular transport factor p115, USO1 homolog (USO1), which were identified with the HeLa cells cDNA library, and the fibrinogen alpha-chain (FGA) that was identified using the human fetal liver cDNA library (Table 1). The dynein light-chain, Tctex-type 1 (DYNLT1) and the inter-alpha-trypsin inhibitor heavy chain 3 (ITIH3) were caught with both libraries, while the filamin B, beta (FLNB) was only fished with the human fetal liver cDNA library.

**Table 1:** Yeast two-hybrid screening with the HrpA middle region (HrpA-M)

Clone #	Interacting host protein	Nucleotide sequence
HeLa cDNA library		
1	SUMO-conjugating enzyme (UBC9)	104–612
2	Dynein light-chain, Tctex-type 1 (DYNLT1)	57–747
3	General vesicular transport factor p115, USO1 homolog (USO1)	653–1109
4	General vesicular transport factor p115, USO1 homolog (USO1)	968–1162
5	SUMO-conjugating enzyme (UBC9)	82–580
6	Inter-alpha-trypsin inhibitor heavy chain 3 (ITIH3)	1713–2289
Human fetal liver cDNA library		
7	Dynein light-chain, Tctex-type 1 (DYNLT1)	40–765
8	Inter-alpha-trypsin inhibitor heavy chain 3 (ITIH3)	1713- 2597
9	Fibrinogen alpha-chain (FGA)	560–1285
10	Filamin B, beta (FLNB)	5748–6168
11	Fibrinogen alpha-chain (FGA)	560–1285

### Specific HrpA-M sub-regions are involved in the interaction with the host proteins

HrpA-M1, HrpA-M2 and HrpA-M3 sub-regions of HrpA-M (Fig. 1D) were cloned into pGBKT7 and challenged with the isolated plasmid clones by two-hybrid system in order to further confirm the interactions, and, at the same time, to try to identify the specific interacting domains (Fig. 3). Results demonstrated distinct interaction patterns of the host proteins with HrpA (Table 2). DYNLT1 exhibited a specific and exclusive interaction with HrpA-M3 (Fig. 3A), which encompasses the HrpA coiled-coil structure (Fig. 1D). HrpA-M3 was also involved in the interaction with USO1, UBC9, ITHI3 and FGA, although these proteins also interacted with other HrpA-M sub-regions (Fig. 3B, D, E, F, respectively). In particular, USO1 interacted with HrpA-M2 and HrpA-M3; UBC9 and FGA interacted with HrpA-M1 and HrpA-M3; ITH3 interacted with all HrpA-M sub-regions (Table 2). In contrast, the HrpA-M1 and HrpA-M2 sub-regions mediated the interaction with FLNB (Fig. 3C and Table 2). These findings hinted at a versatile role for HrpA-M domain.

**Table 2:** Interaction of host proteins with HrpA-M and sub-regions HrpA-M1, HrpA-M2 and HrpA-M3

Interacting host protein	HrpA-M	HrpA-M1	HrpA-M2	HrpA-M3
Dynein light-chain, Tctex-type 1 (DYNLT1)	+ + +	−	−	+ + +
Fibrinogen alpha-chain (FGA)	+ +	+ +	−	+ +
SUMO-conjugating enzyme (UBC9)	+ + +	+ + +	−	+ +
General vesicular transport factor p115, USO1 homolog (USO1)	+ +	−	+ +	+ + +
Inter-alpha-trypsin inhibitor heavy chain 3 (ITIH3)	+ +	+ + +	+ + +	+ + +
Filamin B, beta (FLNB)	+ + +	+ + +	+ + +	−

### The interaction between HrpA-M3 and the DYNLT1 was confirmed by pull-down assay and co-localization experiments in infected HeLa cells

**Fig. 4:**
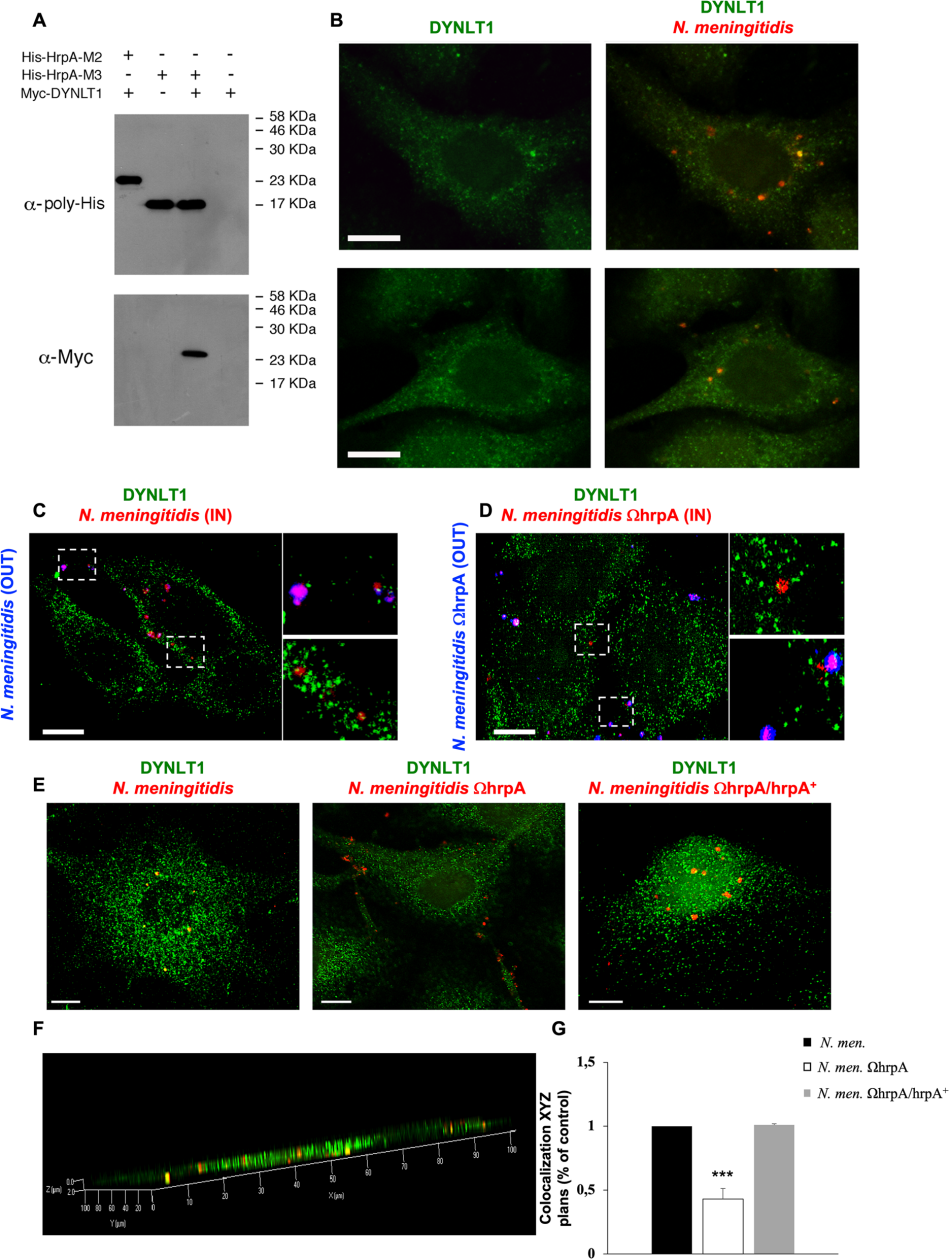
In vitro interaction between HrpA-M3 and the DYNLT1, and co-localization of *N. meningitidis* with DYNLT1 in infected HeLa and HBMEC cells. **A** Interaction between histidine-tagged HrpA-M3 (His-HrpA-M3) and Myc-tagged DYNLT1 (Myc-DYNLT1) was analyzed by pull-down experiments. Purified His-HrpA-M2 or His-HrpA-M3 were bound to Ni–NTA resin and incubated with a HeLa cell lysate overexpressing Myc-DYNLT1. The negative control was represented by Ni–NTA resin alone. Eluted proteins were detected by western blotting using mouse antibodies against poly-His (α-poly-His) or Myc (α-Myc). **B** HeLa cells were infected with *N. meningitidis* strain B1940. After infection, cells were exposed to gentamicin to kill remaining extracellular bacteria, washed to eliminate the majority of dead extracellular bacteria, and analyzed by immunofluorescence microscopy. Green labeled DYNLT1 and red stained *N. meningitidis* are shown in merged images in the panels on the right. Bars, 10 µm. **C, D** HeLa cells were infected with *N. meningitidis* B1940 strain and B1940ΩhrpA derivative mutant. Bacteria were immunostained before permeabilization (blu) and after permeabilization (red) to visualize extracellular (purple) and intracellular (red) bacteria. DYNLT1 was immunostained also after permeabilization and showed in green. White dashed squares show zoomed areas. Bars, 10 µm. **E** HBMEC cells were infected with wild type bacteria, ΩhrpA mutant derivative strain and the *hrpA*-complemented strain B1940 ΩhrpA/hrpA + . DYNLT1 (green) and *N. meningitidis* (red) are shown in merged images. Bars, 10 µm. **F** Example of colocalization between B1940 wild type strain and DYNLT1 in HBMEC cells using the XYZ orthogonal view. **G** The degree of colocalization between the three strains of meningococci and DYNLT1 in HBMEC cells was determined using the XYZ orthogonal view. Values are the mean ± SEM of three different experiments where bacteria present in at least 50 cells/sample were scored. *P < 0.05 **P < 0.01 ***P < 0.001

In the following experiments, we focused on DYNLT1. The interaction between the DYNLT1 and HrpA-M3 was evaluated by pull-down experiments using HrpA-M2 as a negative control (Fig. 4A). His-tagged HrpA-M2 or HrpA-M3 peptides were expressed in bacteria (Additional file 1: Fig. S2E, F) and purified using the Ni–NTA resin (Additional file 1: Fig. S5). Purified proteins were bound to Ni–NTA resin and incubated with lysates of HeLa cells expressing the Myc-tagged DYNLT1. Then the resin was extensively washed and bound proteins were analyzed by Western blotting using mouse antibodies against His or Myc tags. As expected, His-tagged proteins were present in the samples while the myc-tagged DYNLT1 protein was present only when His-tagged HrpA-M3 was used (Fig. 4A, lane 3) but not when incubated with His-tagged HrpA-M2 (Fig. 4A, lane 1) or when it was loaded onto the Ni–NTA resin alone (Fig. 4A, lane 4). This result demonstrated the ability of HrpA-M3 to interact specifically with DYNLT1 in an in vitro system.

Results from previous work suggest that *N. meningitidis* strain B1940, after entering HeLa cells, escape from the internalization vacuole into the cytoplasm [11]. Here we used the same cell infection model to look at possible co-localization between meningococci and the DYNLT1 (Fig. 4B). In these experiments HeLa cells were infected with meningococcal strain B1940 at a MOI of 50 for 1 h. Cells were washed extensively with PBS to remove gentamicin and dead extracellular bacteria and then analyzed by immunofluorescence using specific antibodies to visualize internalized meningococci (red-stained) and DYNLT1 (green-stained). Merged images showed that many intracellular meningococci actually co-localized with DYNLT1 (Fig. 4B). Furthermore, we have performed the staining with an anti-*Neisseria meningitidis* antibody before and after cell permeabilization in order to discriminate intracellular (red) and extracellular bacteria (purple). This analysis was performed in HeLa cells infected with either the B1940 wild type strain (Fig. 4C) or the B1940 $\Omega$hrpA derivative mutant (Fig. 4D) and allowed us to observe that, compared to wild type bacteria, fewer mutant bacteria colocalized with DYNLT1. We also performed this experiment in the more appropriate HBMEC (Human Brain Microvascular Endothelial Cells) model cell line. Indeed, HBMEC are considered an established model to study meningococcal pathogenicity [54]. In these experiments, in addition to the wild type and ΩhrpA mutant B1940 strains we also used the *hrpA*-complemented strain B1940 ΩhrpA/hrpA + (Fig. 4E). Using XYZ orthogonal view we quantified the degree of colocalization in HBMEC (Fig. 4F, G) and we found that only ~ 50% of B1940 ΩhrpA mutant bacteria colocalized with DYNLT1 compared to the wild type bacteria. Interestingly, when HBMEC were infected with *hrpA*-complemented strain we observed a complete rescue of the wild type behavior (Fig. 4G).

### The HrpA-M interface interacting with the DYNLT1 has similarity with viral capsid proteins interacting with the DYNLT1

**Fig. 5:**
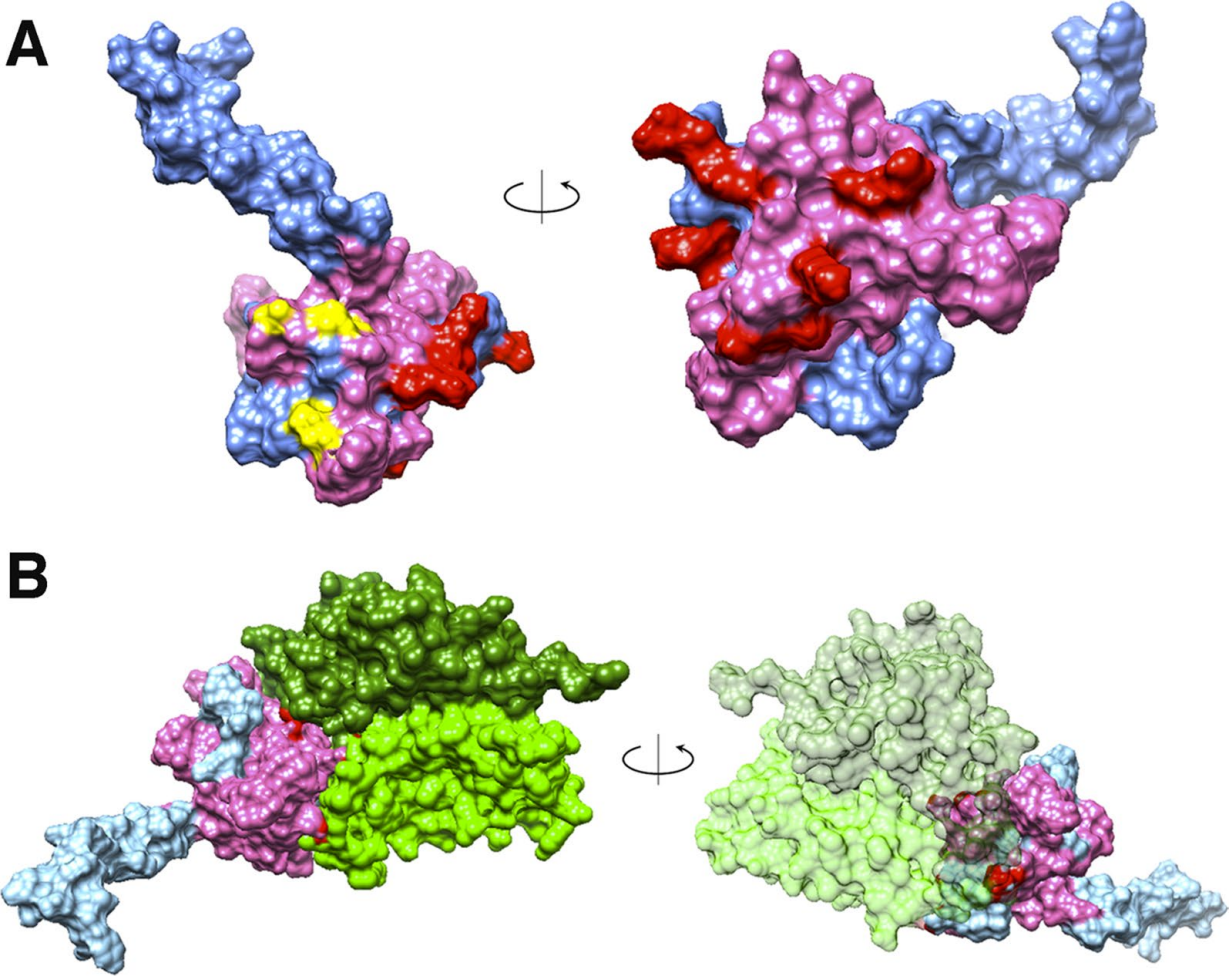
In silico interaction between HrpA-M3 and DYNLT1. **A** HrpA-M3 3D model generated by I-Tasser. The region of homology between HrpA-M3 and viral proteins HHV-1 UL35 and HPV-16 L2 interacting with DYNLT1 is marked in fuchsia. Docking sites with DYNLT1 5jpw crystallographic model or DYNLT1 I-Tasser model are marked in yellow or in red, respectively. The remaining regions of HrpA-M3 are azure. **B** Interaction between HrpA-M3 and DYNLT1 (5jpw crystallographic model) dimer. The two DYNLT1 monomers are marked in light and dark green, respectively. The other features are marked as in **A**

A number of viral proteins are known for their ability to interact with DYNLT1 and to be involved in the intracellular traffic of viral particles [40]. Analysis with the William Pearson’s LALIGN program revealed homology between HrpA-M3 and some of these viral proteins. In particular, HrpA-M3 exhibited extensive similarity with HHV-1 UL35, and HPV-16 L2 capsid proteins, and with cellular CD155 receptor of *Poliovirus*, which are known to interact with DYNLT1 [41–43] (Additional file 1: Fig. S3). Similarity was mostly restricted to the HrpA-M3 region between the amino acid 26 and 100 (corresponding to amino acids 983–1057 of full-length HrpA [NMB1779]). This region of homology to viral proteins is marked in fuchsia in the HrpA-M3 3D model that was obtained by using the I-Tasser package (Fig. 5A).

HrpA-M3 3D model generated by I-Tasser was then used to analyze the in silico interaction with DYNLT1. To this purpose, HrpA-M3 was docked against two pdb files of DYNLT1: crystallographic model of DYNLT1 (5jpw, modified as described in Materials and Methods), and an in silico model of human DYNLT1 homodimer (marked in green) that was generated by I-Tasser (Fig. 5B). Docking simulations were carried out by using PatchDock and refined by using FireDock. The same analysis was carried out with HHV-1 UL35 as a positive control. Results demonstrated that predicted free energies resulting from in silico interaction between HrpA-M3 and DYNLT1 (with both crystallographic and I-Tasser I- DYNLT1 models) and HHV-1 UL35 and DYNLT1 were similar (Additional file 1: Table S2). The analysis of the predicted HrpA-M3/DYNLT1 3D model showed that the HrpA-M3 region of homology to viral HHV-1 UL35 and HPV-16 L2 was mostly restricted to the binding interface with DYNLT1 (Fig. 5B).

### HrpA plays a role in meningococcal survival and intracellular distribution in infected neuronal cells

The homology between HrpA-M3 region and the capsid proteins of neurotropic viruses that hijack the dynein motor for retrograde transport along the axons to gain access and sustain infection in the central nervous system [55], raised the question whether the meningococcus has the ability to interact directly with neuronal cells, and whether HrpA may have a role in this putative interaction. To address this question, we used the NSC34 hybrid cell line derived from the fusion of mouse motor neuron-enriched embryonic spinal cord cells with mouse neuroblastoma cells [56]. Despite the fact that the NSC-34 cell line was derived from mouse, a non-optimal animal model for the study of the infection cycle of the meningococcus, a human-adapted pathogen, we chose these cells because, when subjected to differentiation protocols, they assume behaviors similar to that of primary (motor) neurons in culture [56], and therefore are widely used to investigate neurotoxicity and neurodegenerative disorders in humans [57–62]. In addition, NSC34 cells have been used as a model to investigate the dysfunction of the dynein complex in neurodegenerative disorders [63–65], and hence were believed to be a suitable system to study the interaction between *N. meningitidis* and the dynein motor.

**Fig. 6:**
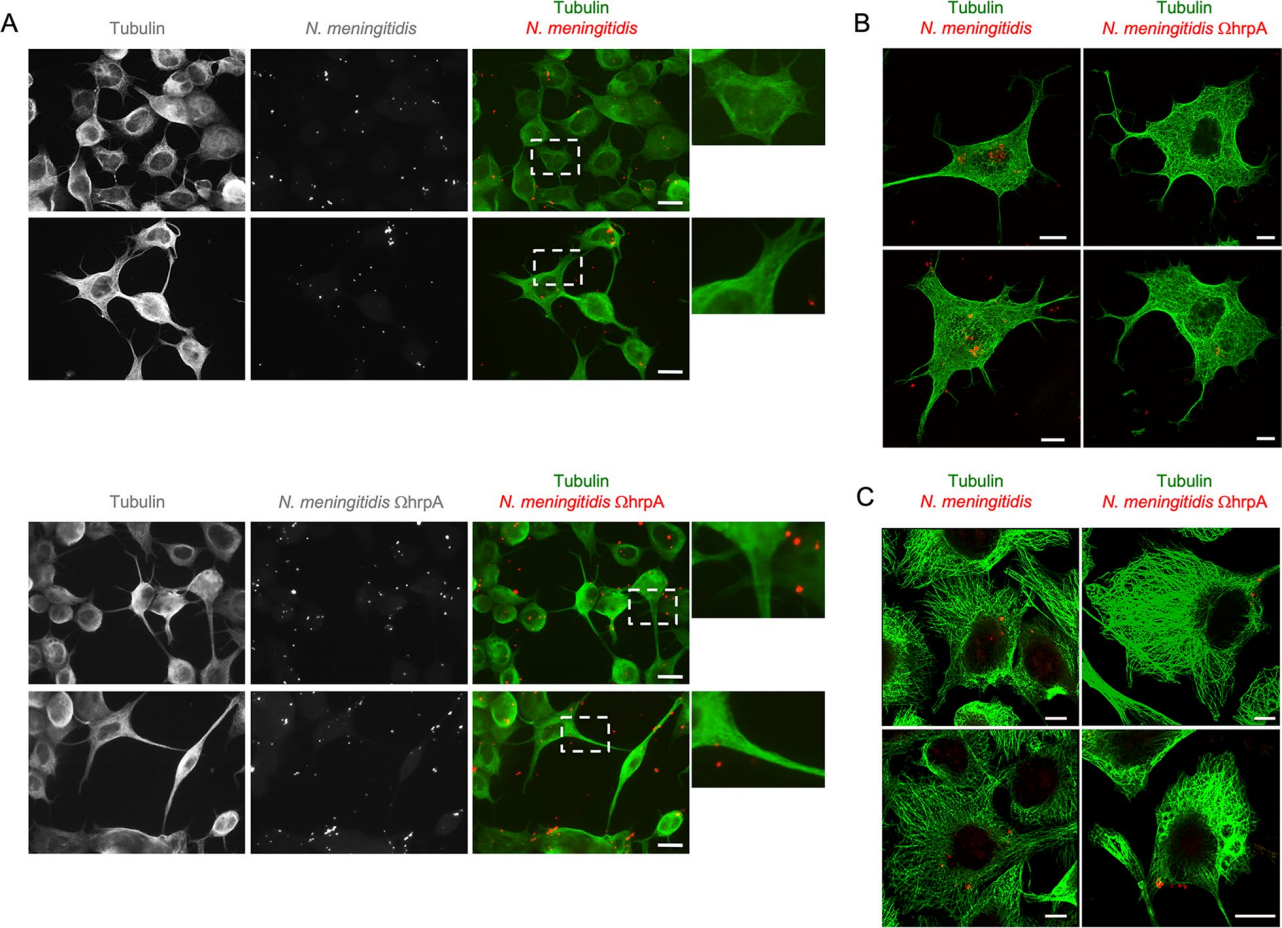
Infection of NSC34 neuronal cells with *N. meningitidis.* **A** NSC34 neuronal cells were infected with either *N. meningitidis* wild type strain B1940 (upper panels) or B1940ΩhrpA derivative mutant (lower panels) as indicated. After infection, cells were exposed to gentamicin to kill remaining extracellular bacteria, washed to eliminate the majority of dead extracellular bacteria, and analyzed by immunofluorescence microscopy. Tubulin (green) and *N. meningitidis* (red) are shown in merged images in the panels on the right. White dashed squares show zoomed areas. Bars, 10 µm. NSC34 (**B**) or HBMEC (**C**) cells were infected with either B1940 wild type or B1940ΩhrpA mutant strains, as indicated, processed as in panel **A**, and then analyzed by confocal laser scanning microscopy. Tubulin (green) and *N. meningitidis* (red) are shown in merged images. Bars, 10 µm

**Fig. 7:**
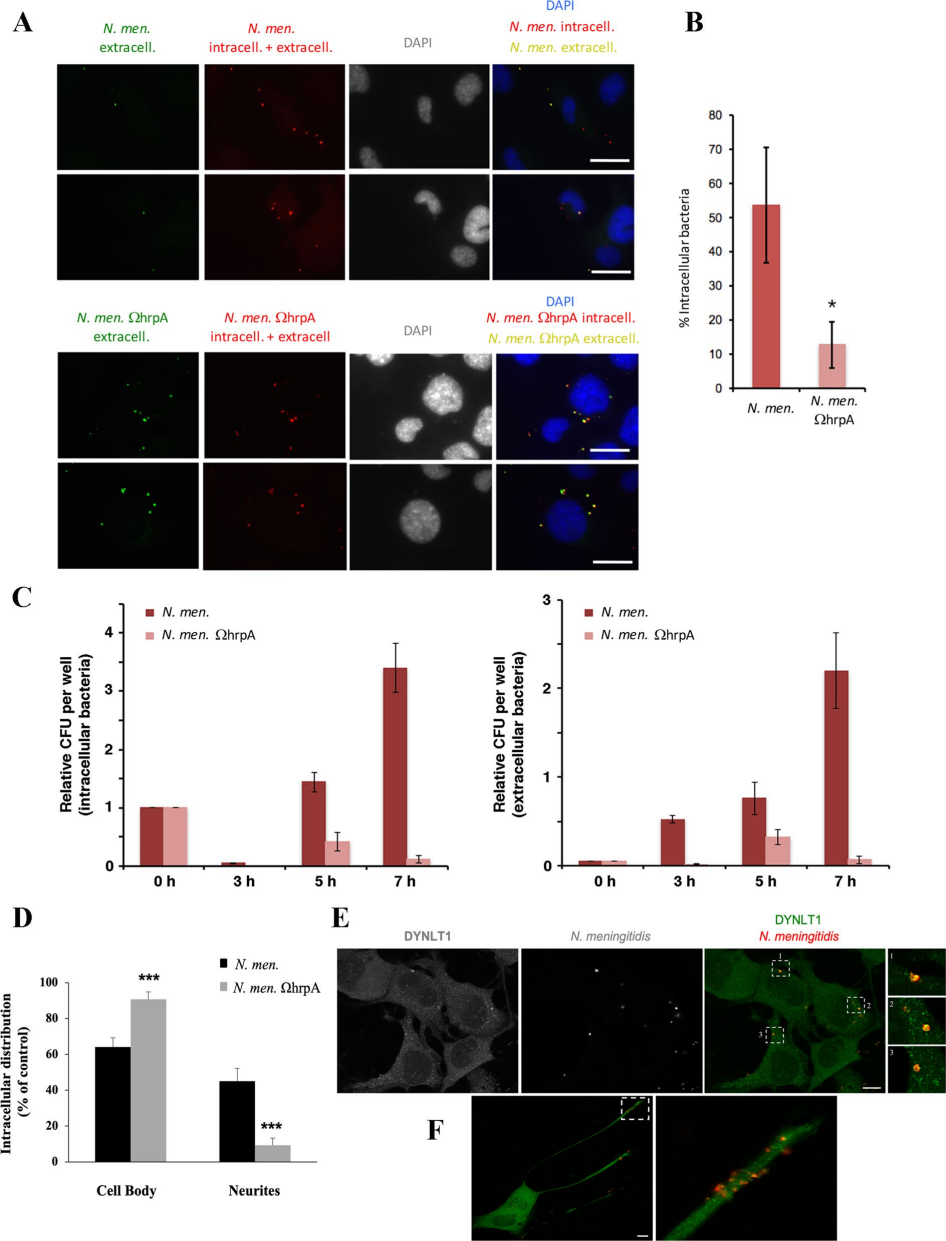
Intracellular abundance and distribution of *N. meningitidis*. **A** NSC34 neuronal cells were infected with either *N. meningitidis* wild type strain B1940 or B1940ΩhrpA derivative mutant as indicated. Immunofluorescence microscopy shows green and red stained extracellular bacteria (yellow in merged image), while intracellular bacteria are only stained in red. Bars,10 µm. **B** Quantification of intracellular wild type and *hrpA*-defective mutant meningococci. Extracellular and intracellular bacteria were counted in at least 50 cells per sample in different fields. Then, we calculated the percentage of intracellular bacteria on the total number of bacteria scored. Data shown are the mean ± SEM of three independent experiments. **C** Results of the gentamicin protection assay. Number of wild type or *hrpA*-defective mutant surviving and growing in NSC-34 cells 3, 5 and 7 h post-infection. Values are the mean ± SEM of three different experiments. *P < 0.05 **P < 0.01 ***P < 0.001. **D** Quantification of the intracellular distribution of wild type and mutant bacteria in cell body and neurites. Measures were obtained by analyzing at least 50 cells/sample in three independent experiments **E**, **F** Immunostaining of NSC-34 neuronal cells with anti-DYNLT1 (green) and with anti-*N. meningitidis* (red) antibodies shows co-localization (yellow) between DYNLT1 and intracellular meningococci and clustering of meningococci in neurites. White dashed boxes indicate zoomed areas on the right. Scale bar: 10 μm

In our experiments NSC34 cells were seeded at low density and grown for 48 h to induce differentiation. Then the cells were infected with the *N. meningitidis* B1940 wild type strain or with the B1940 ΩhrpA derivative mutant [11] at a MOI of 50 for 1 h. Cells were washed gently to eliminate the majority of dead extracellular bacteria after gentamicin treatment, and then analyzed by immunofluorescence microscopy. Figure 6 shows NSC34 cells with neurite-like processes forming a network, and a similar number of meningococci interacting with neuronal cells with either the B1940 strain or the B1940ΩhrpA mutant (Fig. 6A, B). Similarly, when we stained microtubules in HBMEC cells, we found a similar distribution of both wild type and mutant meningococci (Fig. 6C). Then, we used permeabilization protocols to distinguish between adherent extracellular (yellow) and intracellular bacteria (red) (Fig. 7A). We counted extracellular and intracellular bacteria in different microscopy fields to obtain the percentage of intracellular bacteria per cell (Fig. 7B). Significant differences were found between the two strains (Fig. 7A, B). Indeed, more than half of the wild type meningococci (53.57 ± 16.9%) were detected in the intracellular environment, while only a small percentage (12.7 ± 6.7%) of *hrpA*-defective mutant bacteria were observed inside NSC-34 cells (Fig. 7A, B). The differences between the two strains were statistically significant (p-value 4.45208E-05) suggesting that serogroup B meningococci have the ability to infect and survive in neuronal cells, and this ability is dependent on HrpA. These data were confirmed by gentamicin protection assay indicating that 3, 5 and 7 h post-infection there was a consistent number of wild type bacteria surviving and growing in NSC34 cells while only a small number of HrpA mutant bacteria were surviving in these cells (Fig. 7C). In addition, analysis of the intracellular distribution of bacteria revealed that while the wild type bacteria were present at 64% (± 5.3) in cell body and at 45% (± 7.13) in neurites, the mutant bacteria were mostly present in the cell body (91 ± 4%) and only rarely in neurites (9 ± 3.9%) (Fig. 7D). Finally, confocal immunofluorescence microscopy demonstrated that intracellular meningococci and DYNLT1 co-localization was close to 100% (Fig. 7E), with clusters of bacteria often detected in neurites (Fig. 7F).

### DYNLT1 silencing stimulates apoptosis in infected cells

**Fig. 8:**
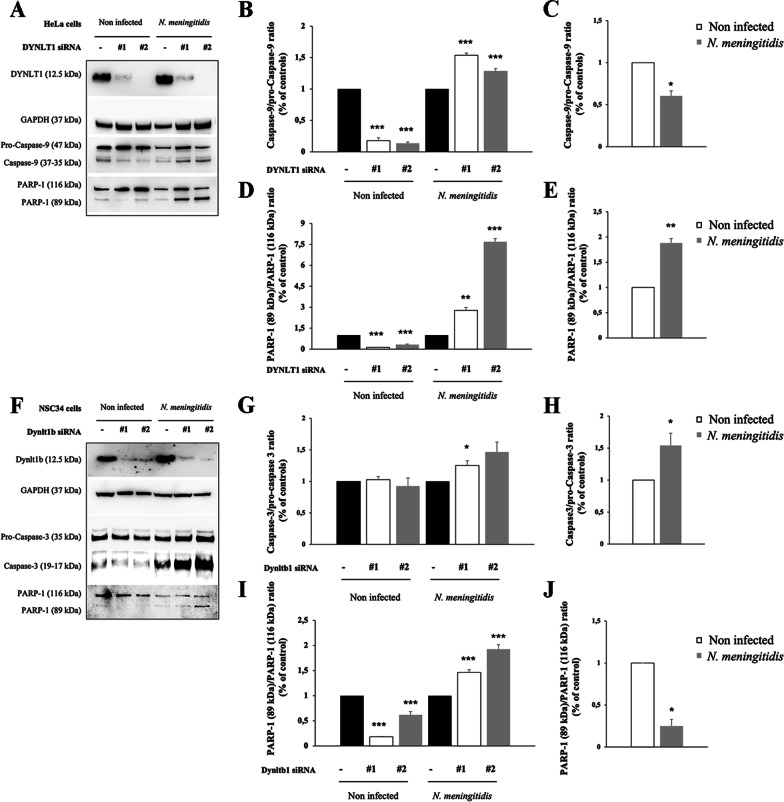
Effects on apoptosis in HeLa and NSC34 cells. Lysates from HeLa (**A**–**E**) and NSC34 (**F**–**J**) cells transfected with control RNA and with two different DYNLT1 siRNAs (indicated as #1 and #2) were subjected to SDS-PAGE and analyzed by immunoblotting using specific antibodies for Caspase-9, Caspase-3 and PARP-1. Antibodies against GAPDH were used to verify loading while antibodies against DYNLT1 were used to check dynein silencing. **B**, **D**, **G**, **I** Signal intensity was quantified by densitometric analysis and reported as fold changes of DYNLT1 siRNA samples compared to their controls. **C**, **E**, **H**, **J** Signal intensity was quantified by densitometric analysis and reported as fold changes of infected samples compared to non-infected cells. Values are the mean ± SEM of at least three independent experiments. *P < 0.05 **P < 0.01 ***P < 0.001

The results of the infection experiments with the B1940 strain or the B1940ΩhrpA mutant showed that HrpA is important for surviving in neuronal cells, and it is a determinant for intracellular distribution of bacteria. As meningococcus has been shown to inhibit apoptosis in a variety of infected cells, including macrophages, umbilical vein endothelial cells and HeLa cells [66–70], we wondered if meningococcus had the ability to do this also in neuronal cells in a similar way to certain neurotropic viruses, and if this ability depended on the interaction between HrpA and DYNLT1. To assess the involvement of DYNLT1/HrpA interaction in these processes, siRNA-mediated silencing of DYNLT1 was carried out, using two different siRNAs, in HeLa cells and in NSC34 neuronal cells, which were then infected with B1940 strain. To monitor apoptosis, we analyzed by Western blot the cleavage of pro-Caspase-9 and PARP-1 in HeLa cells (Fig. 8A) and the cleavage of pro-Caspase-3 and PARP-1 in NSC34 neuronal cells (Fig. 8F). PARP-1 is a substrate of caspases and its cleavage is considered the hallmark of apoptosis [71]. The cleavage of PARP-1 produces two specific fragments: an 89-kD catalytic fragment, that reaches the cytosol, and a 24-kD DNA binding domain (DBD), retained in the nucleus [72]. PARP-1 has a pivotal role in signaling single-strand breaks (SSB) [73] and its cleavage induces accumulation of SSBs, double-strand breaks (DSBs) and, finally, cell apoptotic death [74]. Silencing of DYNLT1 in HeLa cells resulted in suppression of apoptosis, consistently with previous reports demonstrating a role of dynein in apoptosis [75]. Indeed, we observed with both siRNAs in HeLa cells a significant reduction of caspase-9/pro-caspase-9 ratio (83% ± 0.04 and 86% ± 0.018) and a significant decrease of PARP 89 kDa fragment (86% ± 0.08 and 66% ± 0.03) compared to full length of PARP-1 (Fig. 8B, D). To exclude off-target effects we performed a rescue experiment expressing back DYNLT1 after silencing in HeLa cells. We monitored cleavage of PARP-1 and we were able to rescue the apoptotic phenotype (Additional file 1: Fig. S4).

Interestingly, infection of DYLNT1-silenced HeLa cells determined activation of the apoptotic pathway. Indeed, after infection, we observed, with both siRNAs, a significant increase of caspase-9 (to about 1.5 fold ± 0.03 and 1.3 fold ± 0.04) and of 89 kDa PARP-1 fragment (to about 2.7fold ± 0.21 and 7.6fold ± 0.23) compared to infected scrambled cells, used as a control (Fig. 8A, B, D). Nevertheless, we found a significant reduction of caspase-9 and a significant increase of 89 kDa PARP-1 fragment after infection (Fig. 8C, E). Similar results were obtained in DYNLT1 silenced NSC34 cells (Fig. 8F). In particular, we found a significant decrease of PARP-1 cleavage in non-infected silenced cells (about 80% ± 0.01 and 40% ± 0.11) and an increase of caspase-3/pro-caspase-3 ratio (1.25 fold ± 0.12 and 1.46 fold ± 0.27) and of PARP-1 cleavage (1.46 fold ± 0.09 and twofold ± 0.15) in silenced NSC34 cells after infection (Fig. 8G, I). Interestingly, we observed a significant increase of caspase-3/pro-caspase-3 ratio (1.5 fold ± 0.19) after infection (Fig. 8H) and a decrease of 89 kDa PARP-1 fragment after infection (Fig. 8J).

### HrpA activates pyroptosis pathways in HeLa and NSC34 infected cells

**Fig. 9:**
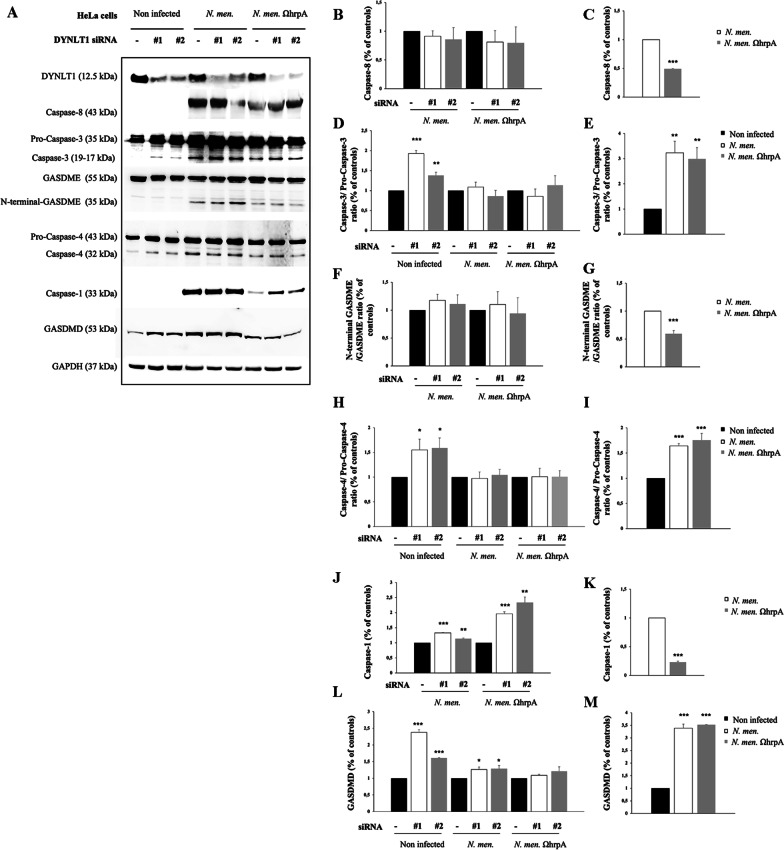
Effects of infection of HeLa cells by *N. meningitidis* B1940 or B1940ΩhrpA strains on apoptosis and pyroptosis markers. **A** Lysates from HeLa cells transfected with control RNA and with two different DYNLT1 siRNAs (indicated as #1 and #2) were subjected to SDS-PAGE and analyzed by immunoblotting using specific antibodies for Caspase-8, Caspase-3, N-terminal Gasdermin E (GSDME), Caspase-4, Caspase-1, and Gasdermin D (GSDMD). Antibodies against GAPDH were used to verify loading while antibodies against DYNLT1 were used to check dynein silencing. **B**, **D**,** F**,** H**,** J**, **L** Signal intensity was quantified by densitometric analysis and reported as fold changes of DYNLT1 siRNA samples compared to their controls. Values are the mean ± SEM of at least three independent experiments. *P < 0.05 **P < 0.01 ***P < 0.001. **C**, **E**,** G**,** I**, **K**, **M** Signal intensity was quantified by densitometric analysis and reported as fold changes of infected samples compared to non-infected cells or compared to wild type if no signal was detected in non-infected cells. Values are the mean ± SEM of at least three independent experiments. *P < 0.05 **P < 0.01 ***P < 0.001

**Fig. 10:**
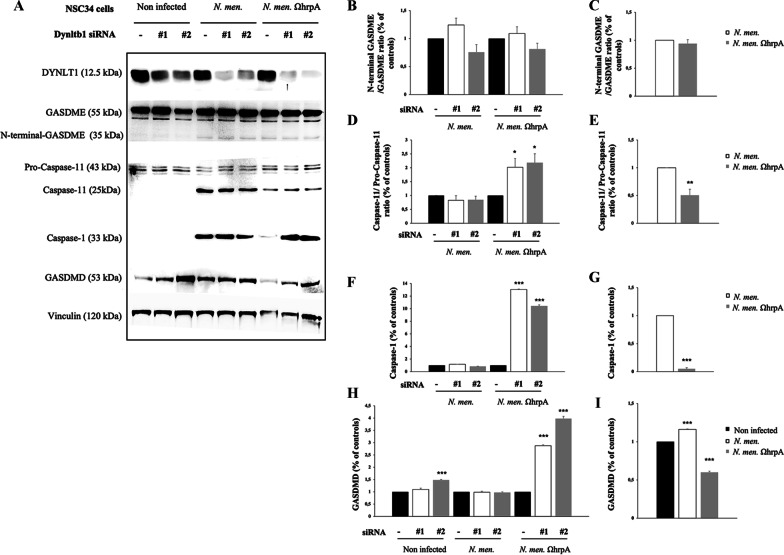
Effects of infection of NSC34 neuronal cells by *N. meningitidis* B1940 or B1940ΩhrpA strains on apoptosis and pyroptosis markers. Lysates from NSC34 cells transfected with control RNA and with two different dynlt1b siRNAs (indicated as #1 and #2) were subjected to SDS-PAGE and analyzed by immunoblotting using specific antibodies for Gasdermin E N-terminal (GSDME), Caspase-11, Caspase-1 and Gasdermin D (GSDMD). Antibodies against GAPDH were used to verify loading while antibodies against DYNLT1 were used to check dynein silencing. **B, D, F, H** Signal intensity was quantified by densitometric analysis and reported as fold changes of DYNLT1 siRNA samples compared to their controls. Values are the mean ± SEM of at least three independent experiments. *P < 0.05 **P < 0.01 ***P < 0.001. **C, E, G, I** Signal intensity was quantified by densitometric analysis and reported as fold changes of infected samples compared to non-infected cells or or compared to wild type if no signal was detected in non-infected cells. Values are the mean ± SEM of at least three independent experiments. *P < 0.05 **P < 0.01 ***P < 0.001

The results of the apoptosis experiments showed an increase of caspase-3 activation after infection in NSC34 cells. Similarly, analysis of caspase-3 in HeLa cells revealed a significant increase in the caspase-3/pro-caspase-3 ratio (90% ± 0.07 and 40% ± 0.08) in silenced non-infected HeLa cells, and a strong increase of the same ratio (threefold ± 0.46) after infection with both meningococci (Fig. 9A, D, E). Moreover, we analyzed caspase-8 and we found that it is only present in infected cells with higher levels in cells infected with wild type bacteria compared to cells infected with *hrpA* mutant bacteria (Fig. 9A, B, C). Notably, caspase-3 is also heavily involved in pyroptosis [73, 74], a form of pro-inflammatory cell death that relies on the caspase family, and it is one of the programmed cell death modes. There are two known pathways: i) classical pathway (or canonical inflammasome) which is dependent on caspase-1; ii) non-classical pathway (or non-canonical inflammasome) which is dependent on caspase-4/5/11. These pathways converge in the activation of Gasdermin D (GSDMD) which occurs by cleavage of the 53 kDa GSDMD to form an N-terminal domain with 31 kDa, thereby forming pyroptosis [76]. In recent years, the caspase-3/ Gasdermin E (GSDME)-dependent pyroptosis signaling pathway has been discovered. Cleaved caspase-3 induces activation of GSDME by translocation of N-terminal domains in the cell membrane, causing cell swelling and rupture, releasing inflammatory factors and damage-associated molecular patterns (DAMPs) [76, 77]. To assess the involvement of DYNLT1/HrpA interaction in these processes, siRNA-mediated silencing of DYNLT1 was carried out, in HeLa cells and in NSC34 neuronal cells, which were then infected with either B1940 strain or B1940ΩhrpA mutant. As shown in Fig. 9A and F, we found that cleaved N-terminal GASDME was undetectable in uninfected cells in contrast to infected cells, and silencing of DYNLT1 was not accompanied by alterations of N-terminal GASDME abundance. However, a significant reduction (40% ± 0.40) of cleaved GASDME in HeLa cells infected with B1940ΩhrpA strain compared to cells infected with the wild type strain was observed (Fig. 9A, G). Similarly, in the NSC34 cell line, GASDME was not observed in non-infected cells but no differences were found before and after dynein silencing or between the two strains (Fig. 10A, B, C).

These results demonstrated that infection can induce activation of pyroptosis by the caspase-3/GASDME pathway in HeLa and in neuronal cells. In particular, in HeLa cells the infection with B1940 strain induced an increase of the N-terminal GASDME fragment levels compared to B1940ΩhrpA mutant.

Previous work demonstrated that *N. meningitidis* induces activation of pyroptosis by the canonical route [78]. To understand if in HeLa and in NSC-34 cells pyroptosis activation occurs by the classical or by non-canonical pathways after infection, we also analyzed caspase-1 cleavage and GASDMD expression in both cell lines, and cleavage of caspase-4 and caspase-11, respectively in human HeLa and mouse NSC34 cells (Figs. 9A and 10A).

Interestingly, we observed a significant increase of caspase-4/pro-caspase-4 ratio in non-infected DYNLT1 silenced HeLa cells (Fig. 9A, H). Moreover, the caspase-4/pro-caspase-4 ratio was increased also in HeLa cells after infection with either B1940 or B1940ΩhrpA compared to non- infected cells (~ 50% ± 0.20) (Fig. 9A, I). Furthermore, cleaved caspase-11 was not detectable in uninfected NSC34 cells, but we found a significant increase in silenced cells infected with B1940ΩhrpA mutant (Fig. 10A, D). Nevertheless, comparing cleaved caspase-11 levels in cells expressing DYNLT1 and infected with either B1940 strain or B1940ΩhrpA mutant, we observed a significant decrease (50% ± 0.20) of the caspase-11/pro-caspase-11 ratio in cells infected with the mutant (Fig. 10A, E).

Moreover, we found that cleaved caspase-1 was not detectable in uninfected cells, while, consistently with previous findings [78], it could be clearly detectable in both HeLa and in NSC34 cells infected with the B1940 strain (Fig. 9A and Fig. 10A). Notably, it was demonstrated that active caspase-1 is predominantly a transient species of about 33 kDa, [79], as we found DYNLT1 silencing resulted in further moderate increase of caspase-1 activation in B1940-infected cells (Fig. 9A, J). Specifically, in HeLa cells such an increase was 35% ± 0.01% and 15% ± 0.02 (Fig. 9F). It may be also interesting to note that caspase-1 activation was severely reduced (80% ± 0.018 in HeLa cells, and 99. 95% ± 0.004 in NSC34 cells) by genetic inactivation of *hrpA* in the B1940ΩhrpA mutant (Fig. 9K and Fig. 10G), and that DYNLT1 silencing determined a dramatic increase in the amounts of cleaved caspase-1 in both HeLa cells (twofold ± 0.1) and NSC34 cells (tenfold ± 0.01) infected with this mutant (Fig. 9J and Fig. 10F).

The pattern of GASDMD expression approximated that of caspase-1, although we could not see the cleavage of this protein after 24 h of infection. Furthermore, the expression of GASDMD was detectable in full-length form in all cells, either infected or non-infected*.* Specifically, in NSC34 cells, expression of GASDMD was significantly higher in B1940-infected cells than in B1940ΩhrpA-infected (Fig. 10I), while in B1940- or B1940ΩhrpA-infected HeLa cells it was similar (Fig. 9M). DYNLT1 silencing determined an increase of GASDMD expression both in uninfected HeLa cells (1.8 fold) and NSC34 cells (40% ± 0.02), and, especially, in NSC34 cells infected with B1940ΩhrpA (3.5 fold ± 0.09) (Figs. 9L and 10H).

**Fig. 11:**
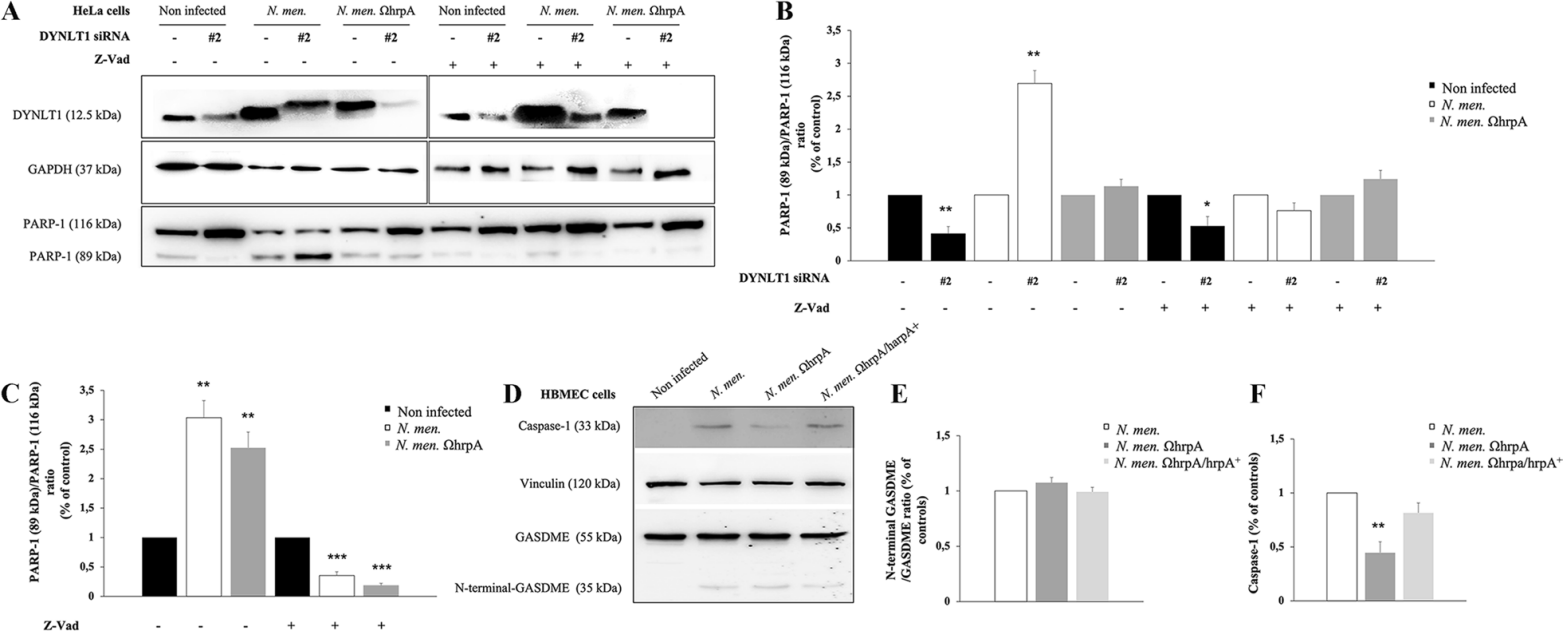
Effects of infection on activation of pyroptotic markers in HBMEC cells and of caspase inhibitor Z-VAD on infected HeLa cells. **A** Lysates from HeLa cells, transfected with control RNA and with DYNLT1 siRNA (indicated as #2), and treated for 24 h with Z-VAD, were subjected to SDS-PAGE and immunoblotting using a specific antibody for PARP-1. Antibodies against GAPDH were used to verify loading while antibodies against DYNLT1 were used to check dynein silencing. **B** Signal intensity was quantified by densitometric analysis and reported as fold changes of DYNLT1 siRNA samples compared to their controls. Values are mean ± SEM of at least three independent experiments. **C** Signal intensity was quantified by densitometric analysis and reported as fold changes of infected samples compared to non-infected cells. Values are the mean ± SEM of at least three independent experiments. *P < 0.05 **P < 0.01 ***P < 0.001. **D** Lysates from HBMEC cells were subjected to SDS-PAGE and analyzed by immunoblotting using specific antibodies for Gasdermin E N-terminal (GSDME) and Caspase-1. Antibodies against vinculin were used to verify loading. **E** Signal intensity was quantified by densitometric analysis and reported as fold changes of infected samples compared to non-infected cells. Values are the mean ± SEM of at least three independent experiments. *P < 0.05 **P < 0.01 ***P < 0.001

Furthermore, we analyzed if the effect of *N. meningitidis* on cell death is able to overcome the effect of the Z-VAD caspase inhibitor (Fig. 11). For this purpose, we evaluated the cleavage of PARP-1 in infected HeLa cells silenced for DYNLT1 (Fig. 11A). Interestingly, we found that the cleavage of PARP-1 is increased (threefold ± 0.19) in cells infected with wild type strain compared to cells infected with mutant, and that Z-VAD is able to inhibit this effect (Fig. 11A, B). Interestingly, infection with both meningococci induced an increase in apoptosis activation and is not able to overcome the effect of the caspase inhibitor (Fig. 11C).

Finally, we evaluated the activation of pyroptosis also in B1940- or B1940ΩhrpA-infected HBMEC cells and we found that cleavage of GASDME is similarly induced by wild type and mutant bacteria (Fig. 11D, E). In contrast, we observed an increase of caspase-1 cleavage after wild type infection compared to mutant and rescue of wild type phenotype after B1940ΩhrpA/hrpA + complemented strain infection (Fig. 11D, F).

## Discussion

The data here reported support the view that the meningococcus-specific HrpA, as well as other large proteins secreted by two-partner secretion systems, represents a class of multitasking proteins with critical roles in bacteria-bacteria and bacteria-host interactions [80]. The multitasking nature of these proteins, i.e., their ability to execute two or more biological functions, is a reliable and convenient way to accomplish many complex and diverse functions with a very limited amount of genetic information. Many functions have been ascribed to HrpA, including contact-dependent inhibition, adhesion to epithelial cells, intracellular escape, immune evasion, and biofilm formation [80]. In this study we attempted to dissect the diverse functions of HrpA by considering the multi-domain nature of this protein, and the predicted structural features of each domain (Fig. 1). By using this approach, we confirmed the Mn^2+^-dependent hemolytic activity of HrpA against human erythrocytes [11], and demonstrated that this activity is confined to the C-terminal region of HrpA (HrpA-C) spanning an FhaB domain (Fig. 2A). This hemolytic activity was related to the ability of HrpA to mediate the escape of the meningococci from the vacuole in epithelial host cells [11]. The FhaB domain of HrpA also contains an SseC domain (Fig. 1A). In *Salmonella enterica*, SseB, SseC and SseD are translocon proteins of the Salmonella pathogenicity island 2 type III secretion system (SPI2-TTSS), and SseC function to insert into the phagosomal membrane to facilitate protein translocation from intraphagosomal bacterial cells into the host cell [53].

The Mn^2+^-dependence of the HrpA-C lysin enlightens the importance of this metal ion in meningococcal metabolism and virulence. It should be noted, however, that in our assay HrpA-C hemolytic activity was induced by millimolar concentrations of manganese ion, well above the normal nanomolar ranges in body fluids [81] or micromolar ranges in brain (20–53 µM) [82]. It is possible, however, that much higher levels might be locally reached in certain microenvironments and/or conditions. High levels of manganese could be reached, for instance, also in proximity of the phagosomal membrane as a consequence of the activity of NRAMP1 and NRAMP2 metal:H^+^ antiports, which play key roles in nutritional immunity by extruding manganese and other essential metals from the phagosome to halt bacterial growth [83]. High manganese levels could be also locally attained on the meningococcal surfaces due to the activity of the MntX transporter [84].

The results of the yeast two-hybrid screening led to the identification of a number of putative host proteins, each interacting with specific subregions of HrpA-M (Tables 1 and 2, and Fig. 3). Some of these interacting proteins play key roles in cytoskeleton organization (FLNB), vesicular traffic (DYNLT1, USO1), and SUMOylation (UBC9) suggesting an interaction of meningococci with these cellular functions during the intracellular phase of the infection cycle. This finding is consistent with a study demonstrating that meningococci have the ability to subvert the polarized organization and intracellular trafficking as a strategy to cross the epithelial nasopharyngeal barrier [85]. Filamin, a class of actin filaments-binding proteins, is also targeted by *S. enterica* SPI2-TTSS effectors SspH2 and SseI, and this interaction was shown to reduce or remodel vacuole-associated actin polymerization induced by intracellular bacteria [86]. DYNLT1 is a dynein light chain that forms homodimers that function as a clump to anchor several viral capsid and cellular proteins to the dynein motor on microtubule filaments [55]. USO1 is a peripheral membrane protein that recycles between the cytosol and the Golgi apparatus during interphase, and is required for transport from the endoplasmic reticulum to the cis/medial Golgi compartments [87]. UBC9 is the ubiquitin conjugating enzyme 9, which is involved in SUMO pathway, the most studied ubiquitin-like pathway that regulates a wide range of cellular events [88]. Similar to ubiquitin, the SUMO pathway is also a target for pathogens. For instance, several secreted pore-forming toxins including *Listeria monocytogenes* listeriolysin O (LLO), *Clostridium perfringens* perfringolysin O (PFO), and *Streptococcus pneumoniae* pneumolysin (PLY) are able to trigger UBC9 degradation [89, 90]. Other putative HrpA-M interactors may be more relevant to other steps of meningococcal pathogenesis, including FGA and ITIH3. ITIH3 belongs to a family of plasma protease inhibitors that contribute to extracellular matrix stability by covalent linkage to hyaluronans, and play a particularly important role in inflammation and carcinogenesis [91]. The putative interaction with ITIH3 might thus be relevant to the interaction of meningococci with the extracellular matrix and vessels, while the interaction with FGA might be related to the ability of meningococci to interfere with components of the coagulation cascade [2]. All these aspects will require further investigation.

In this study we focused on the interaction with DYNLT1. The interaction was confirmed by in vitro pull-down assays and immunofluorescence microscopy experiments showing co-localization of *N. meningitidis* with DYNLT1 in infected HeLa and HBMEC cells (Fig. 4). Both the yeast two-hybrid system (Table 2, and Fig. 3) and in vitro pull-down assays (Fig. 4A) were also instrumental to map the domain of HrpA-M interacting with DYNLT1 to a region (HrpA-M3) characterized by a coiled coil structure (Fig. 1). Protein homology analysis and in silico modeling demonstrated that the interface of HrpA-M3 interacting with the DYNLT1 has extensive similarity with HHV-1 UL35, and HPV-16 L2 capsid proteins, and with cellular CD155 receptor of *Poliovirus*, which are known to interact with the DYNLT1 (Fig. 5). This finding is worthy of note because hijacking the dynein motor by numerous viruses has been postulated to be a general mechanism for virus delivery near the cell nucleus replication site [92, 93]. Similarly, *N. meningitidis* might exploit the dynein motor to move more easily into the crowded cytoplasm, heading towards the microtubule-organizing center (MTOC), the Golgi and the cell nucleus. This hypothesis seems to be also consistent with the ability of HrpA-M to interact in the yeast two-hybrid system with the golgin USO1, and UBC9 that shuttles between the cytoplasm and the nucleus [94]. Future work will be aimed at confirming and elucidating the possible biological role of these interactions in the context of the meningococcal infection and disease. In this regard, it is worthy of noticing that several neurotropic viruses, including the HHV-1 and *Poliovirus*, exploit the dynein motor for retrograde transport along the axons to gain access and sustain infection in the central nervous system [55]. The possibility of a neuronal route in the meningococcal infection is intriguing, and seems to be supported by the evidence that in intranasally infected humanized CD46 mice meningococci can gain direct access to the meninges and the central nervous system via the olfactory nerves that connect the nasal cavity to the brain through the cribriform plate of the ethmoid, even in the absence of bacteraemia [95].

Our findings that *N. meningitidis* is able to effectively infect and survive in neuronal cells (Fig. 6), and that this ability is dependent on HrpA, which establishes a direct protein–protein interaction with DYNLTI (Fig. 7) in these cells, seem to support the hypothesis of a possible neuronal route of infection. About this point, it is important to consider that, in contrast to axon, dendrites exhibit mixed microtubules polarity with minus-end-out and plus-end-out microtubules. This means that while in the axon dynein motors are important for transfer of molecules, organelles and hitch-hiking infectious agents from the periphery to the cell body, the same motors are important for cargoes to get to dendrites. Indeed, dynein-driven transport to dendrites has been described [96]. Therefore, dynein could be important to get to the cell body and near the nucleus but also to get to dendrites, depending on the type of neuron used, suggesting that the HrpA interaction with dynein could be fundamental for *N. meningitidis* spreading inside the neurons. Indeed, the marked neurotropism of this microorganism was confirmed in a mouse model of meningitis showing preferential localization of *N. meningitidis* in the *corpus callosum* [97]. Furthermore, the frequent neurological sequelae in patients who survive meningococcal meningitis are well known, including focal deficits, hearing loss, cognitive impairment and epilepsy [98].

Notably, DYNLT1 silencing stimulated apoptosis in infected HeLa and NSC34 neuronal cells (Fig. 8). Apoptosis is a mechanism for limiting the intracellular multiplication of neurotropic pathogens, and some viruses and pathogenic bacteria have evolved strategies to modulate this process in neural cells [99–101]. Moreover, during an infection, apoptosis in cells of the immune system may be beneficial to the host by dampening inflammation and tissue damage [102]. There is evidence that *N. meningitidis* may suppress apoptosis in a variety of cells [66, 67, 69, 70], although pro- or anti-apoptotic effects may be dependent on the virulence of infecting strains [103], tissue, cell type and microenvironment [104].

**Fig. 12:**
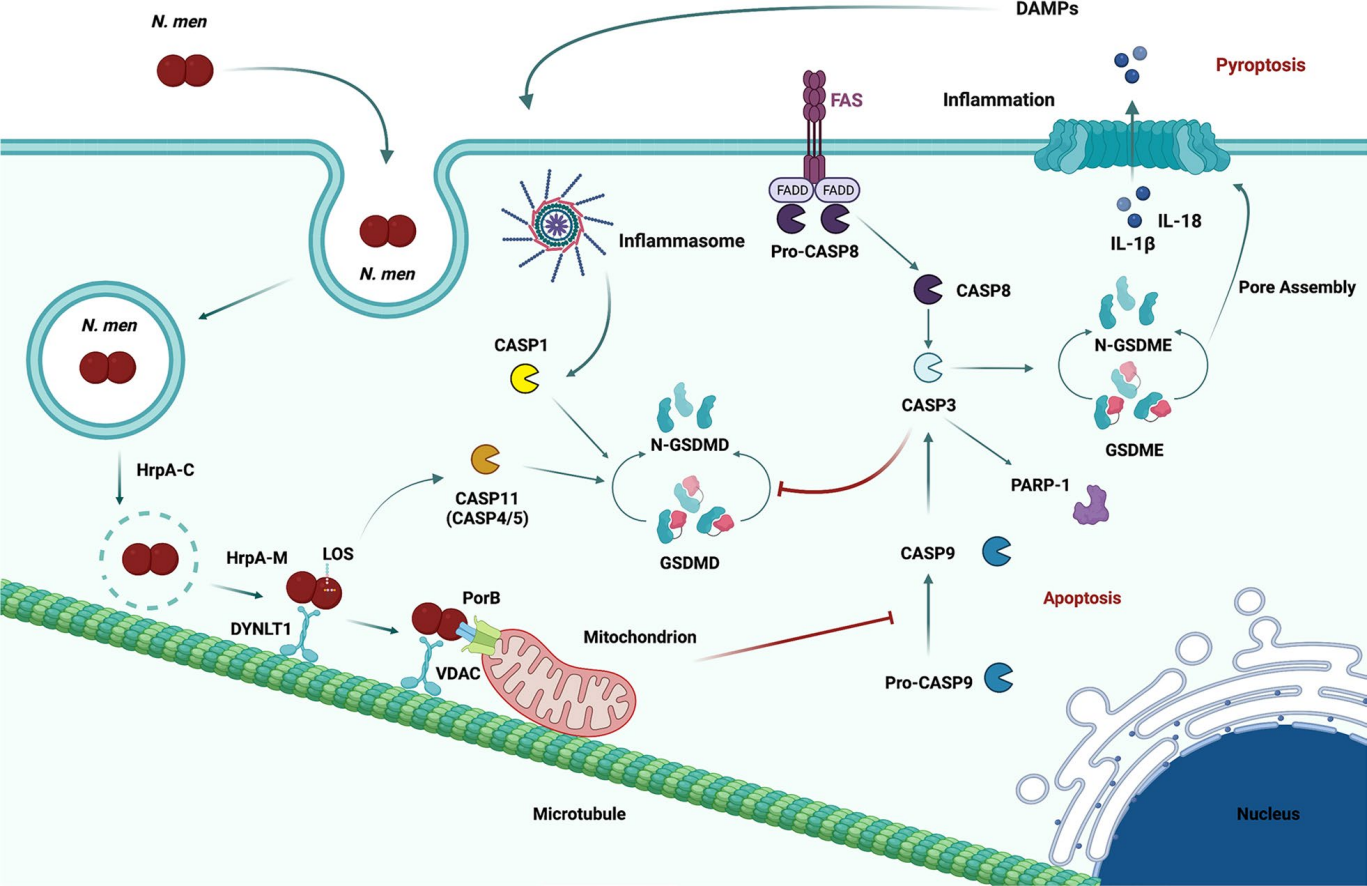
Proposed mechanism of apoptosis inhibition and pyroptosis activation. After *N. meningitidis* infection, HrpA-DYNLT1 interaction allows the translocation of PorB to the mitochondria and its interaction with the voltage-dependent anion-selective channel (VDAC). This interaction determines inhibition of pro-caspase-9 cleavage and of activation of downstream pathway until PARP-1 and, hence, inhibition of apoptosis. *N. meningitidis* can expose the cytosolic lipooligosaccharide (LOS) and induce the non-canonical inflammasome pathway determining cleavage of caspase-11 in mouse and caspase-4/5 in human. Gasdermin D (GSDMD) is an effector of pyroptosis and, after cleavage, its N-terminal domain constitutes a membrane pore to favor secretion of inflammatory mediators. In the proposed model, GSDMD is inhibited by high caspase-3 levels which are involved, instead, in the activation of Gasdermin E (GSDME). Like the GSDMD, GSDME is activated by N-terminal cleavage determining membrane pore assembly to mediate efflux of activate interleukins and induce inflammation and pyroptosis. Damage-associated molecular patterns (DAMPs) derived by dead cells induce activation of inflammasome with consequent caspase-1 activation. Also in this case the effect is aborted as a consequence of the caspase- 3 inhibition on GASDMD. Created with BioRender.com

Stabilization of the mitochondrial membrane by meningococcal porin PorB, which acts as an inhibitor of apoptosis via the intrinsic pathway, is a proposed anti-apoptotic mechanism [66–68]. During infection PorB is progressively translocated to the mitochondria through an unknown mechanism, and once there it prevents mitochondrial membrane depolarization by interacting with voltage-dependent anion-selective channel (VDAC) and possibly by interfering of contact sites between the inner and outer mitochondrial membranes [66, 68]. The interaction between HrpA and DYNLT1 could favor translocation through several mechanisms, for example, either facilitating the contact between bacteria and mitochondria on the tubulin cytoskeleton, or promoting the contact between mitochondria and meningococcal spontaneous outer membrane vesicles (OMV), which carry large amounts of PorB and also contain TPS proteins [105], and/or subsequent PorB translocation. Moreover, there is some evidence that DYNLT1 may be associated with VDAC on mitochondria [106, 107]), and modulate cell death [108] (Fig. 12).

Notably, both in HeLa and NSC34 cells we observed a very strong increase of caspase-3 after infection and caspase-3 is also heavily involved in pyroptosis [73, 74]. We supposed that this increase could be due to extrinsic pathway activation, and it is mediated by caspase-8 (Fig. 9 and Fig. 12). For this reason, we have also analyzed the activation of pyroptosis in HeLa and NSC34 cells and its eventual relationship with DYNLT1 protein. As shown in Fig. 12, we demonstrated that pyroptosis is activated by caspase-3 dependent cleavage of GSDME [76]. Indeed, in HeLa, HBMEC and NSC34 cells we found that GSDME was cleaved to produce the N-terminal fragment only after infection (Figs. 9, 10, 11). It is reasonable to assume that induction of pyroptosis triggers DAMP secretion, which, in turn, strengthens the activation of the inflammasome (Fig. 12). Consistent with this hypothesis, cleaved caspase-1 occurred only in infected cells (Figs. 9, 10, 11). In particular, we observed that the canonical pyroptosis pathway was induced by the wild type strain B1940 and to a much lesser extent by the B1940ΩhrpA derivative mutant in all cell lines, as demonstrated by the analysis of caspase-1 activation (Figs. 9, 10, 11). Importantly, caspase-1 activation in mutant strain was reduced but rescued in the complemented strain (Fig. 11). Furthermore, since most wild type B1940 meningococci, unlike B1940ΩhrpA mutant bacteria, gain the cytosol [11], our finding may also indicate that pyroptosis could be also induced by exposure to cytosolic lipooligosaccharide (LOS) through the non-canonical inflammasome pathway (Fig. 12).

The non-canonical inflammasome involves direct recognition of cytosolic LPS/LOS by the caspase recruitment domains (CARD) of caspase-4 and caspase-5 in humans and caspase-11 in mice [76]. Indeed, we observed strong caspase-11 activation only after infection mostly of wild type B1940 meningococci. Instead, the occurrence of cleaved caspase-4 basal levels also in uninfected cells could indicate that HeLa cells are not the proper cellular model for studying this route probably for their human papillomavirus (HPV) positivity [109]. The caspase-1 and caspase-11 pathways should converge into the promotion of the cleavage of GASDMD. Under these conditions, the N-terminal fragments of GASDMD oligomerize to form membrane pores, and, at the same time, triggers nucleotide-binding oligomerization domain–like receptor pyrin domain–containing-3 (NLRP3)–dependent caspase-1/-11 activation, and, in turn, the proteolytic activation of IL-1β and IL-18 [110]. Surprisingly, we did not find activation of GSDMD, but only full-length expression indicating that pyroptosis activation is independent of this route (Fig. 12). Interestingly, it was demonstrated that caspase-3 suppresses GSDMD dependent cell lysis during extrinsic apoptosis [111] (Fig. 12).

In light of this evidence, we may conclude that the study of the multi-tasking HrpA allowed us to glimpse complex interactions that may occur during meningococcal infection of host cells. Hrpa-C enables the bacteria to gain the cytosol, thereby activating the non-canonical inflammasome pathway via LOS exposure/shedding, while, by anchoring the bacteria to the dynein motor, HrpA-M may facilitate the interaction of meningococci with mitochondria, PorB translocation onto mitochondria and suppression of the intrinsic apoptotic pathway. In this complex balance between apoptotic and pyroptotic pathways, the caspase-3/ GSDME-dependent pyroptosis pathway seems to prevail leading to GSDME activation with possible release of inflammatory factors and DAMPs that, in turn, may stimulate the caspase-1-dependent canonical inflammasome pathway. The balance between apoptotic and pyroptotic pathways is heavily affected by HrpA, as also demonstrated by the *hrpA*-defective strain that, in DYNLT1-silenced cells, behaved similar to the wild type strain in terms of the ability to activate the capsase-11- and caspase-1-dependent inflammasome pathways possibly because it gains the cytosol following the interruption of the endocytic pathway. On a clinical point of view, induction of caspase-3 / GSDME-dependent pyroptosis may well be involved in the inflammation characteristic of invasive meningococcal disease [112]. If these mechanisms are confirmed in future studies, they could open new avenues for the treatment of invasive meningococcal infection.

## Conclusions

In summary, our studies demonstrated that HrpA is fundamental for the ability of *N. meningitidis* to infect neuronal cells and to survive in these cells. This mechanism is mediated by the direct interaction between HrpA and DYNLT1, suggesting that *N. meningitidis* spreading inside the neurons is dependent on this protein–protein interaction. Finally, we found that HrpA affects apoptotic and pyroptotic pathways and this knowledge could provide new treatment options against meningococcal infection in the future.

## Supplementary Information

**Additional file 1.** HrpA anchors meningococci to the dynein motor and affects the balance between apoptosis and pyroptosis.

## Data Availability

The data presented in the study are included in the article and supplementary material.

## Abbreviations

CdiA: Contact-dependent growth inhibition A
DAMPs: Damage-associated molecular patterns
DBD: DNA binding domain
DYNLT1: Dynein light-chain, Tctex-type 1
FBS: Foetal bovine serum
FGA: Fibrinogen alpha-chain
FHA: Filamentous hemagglutinin
FLNB: Filamin B
GAPDH: Glycerol-3-Phosphate-Dehydrogenase
GSDMD: Gasdermin D
GSDME: Gasdermin E
HBE cells: Human bronchial epithelial cells
HHV-1: *Human herpesvirus-1*
His: Hystidine
HpmA: Calcium-independent hemolysin of *Proteus mirabilis*
HPV-16: *Human papillomavirus-16*
HRP: Horseradish peroxidase
HrpA: Hemagglutinin/hemolysin-related proteins A
HrpA-C: C-terminal region of HrpA
HrpA-M: Middle region of HrpA
HrpA-N: N-terminal region of HrpA
IgA1: Human immunoglobulin A1
IORF: Small interfering open reading frame
ITIH3: Inter-alpha-trypsin inhibitor heavy chain 3
LB medium: Luria Bertani medium
LOS: Lipooligosaccharide
LPS: Lipopolysaccharide
MIC: Minimal inhibitory concentration
MntX transporter: Manganese-exporters
MOI: Multiplicity of infection
Nramp protein: Natural-resistance-associated macrophage protein
Opa adhesin: Phase-variable opacity adhesin
ORF: Open reading frame
PARP-1: Poly(ADP-ribose) polymerase-1
PBS: Phosphate-buffered saline
PorB: Porin
SD medium: Synthetic dropout medium
ShlA: Calcium-independent hemolysin of *Serratia marcescens*
SPI2-TTSS: Salmonella pathogenicity island 2-type three secretion system
SSB: Single-strand breaks
Sse: Secretion system effector
TPS system: Two-partner secretion system
TpsB: Channel-forming β-barrel activator/transporter protein
USO1: Yeast homolog of human p115
VDAC: Voltage-dependent anion-selective channel
