# Supplementary material for: HrpA anchors meningococci to the dynein motor and affects the balance between apoptosis and pyroptosis
Source: J Biomed Sci. 2022 Jun 28;29:45. doi: 10.1186/s12929-022-00829-8 (PMC9241232; doi:10.1186/s12929-022-00829-8)
Supplement: Supplementary file 1 — Additional file 1. HrpA anchors meningococci to the dynein motor and affects the balance between apoptosis and pyroptosis. [file 12929_2022_829_MOESM1_ESM.pdf]

HrpA anchors meningococci to the dynein motor and affects the balance between apoptosis and pyroptosis

Adelfia Talà[1§], Flora Guerra[1§], Matteo Calcagnile[1°], Roberta Romano[1°], Silvia Caterina Resta[1], Aurora Paiano[1], Mario Chiariello[2,3], Graziano Pizzolante[1], Cecilia Bucci[1*] and Pietro Alifano[1*]

[1]Department of Biological and Environmental Sciences and Technologies (DiSTeBA) University of Salento, Via Provinciale Monteroni n. 165, 73100 Lecce, Italy
[2]Core Research Laboratory-Siena, Institute for Cancer Research and Prevention (ISPRO), 53100 Siena, Italy.
[3]Institute of Clinical Physiology (IFC), National Research Council (CNR), 53100 Siena, Italy.

§ These authors equally contributed to the work

°These authors equally contributed to the work

Supplementary Material

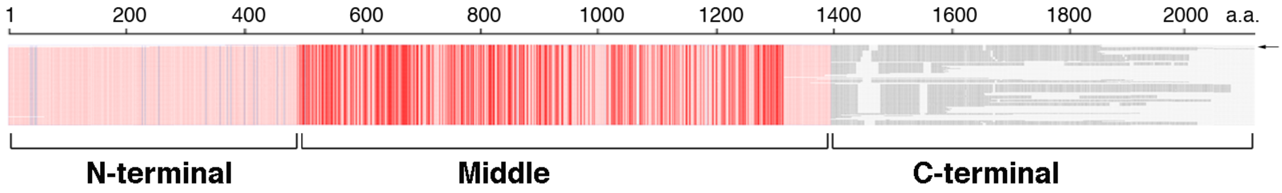

Fig. S1 ESPript multiple alignment and clustering of meningococcal TpsA proteins.

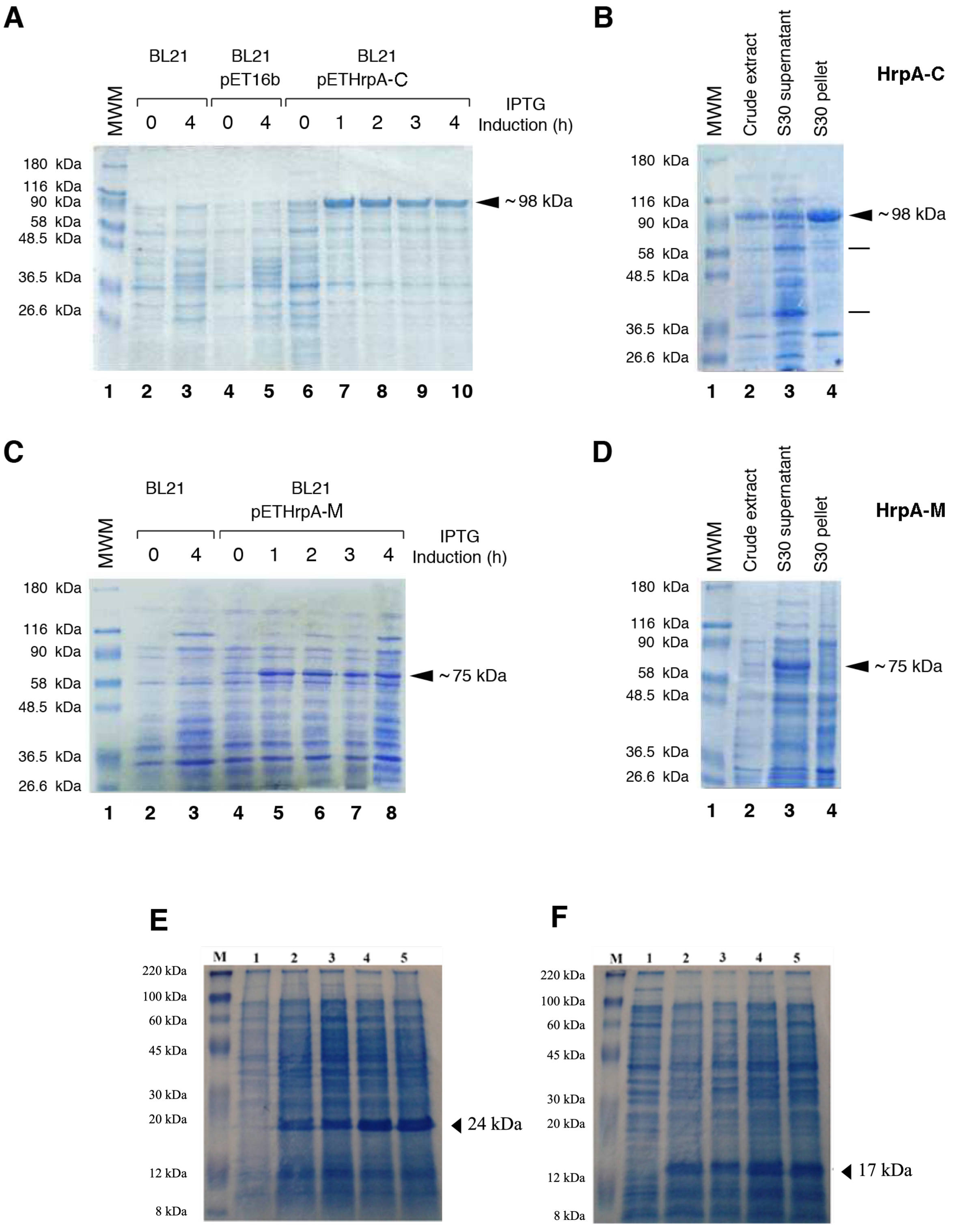

Fig. S2 Expression of recombinant HrpA regions in *E. coli*. **A** Expression of recombinant HrpA C-terminal region (HrpA-C) upon IPTG induction in *E. coli* BL21 (DE3). Crude protein extracts from: BL21 (DE3) cells after 0 or 4 h of IPTG induction (lanes 2 and 3, respectively); BL21 (DE3) cells harboring pET16b vector after 0 or 4 h of IPTG induction (lanes 4 and 5, respectively); BL21 (DE3) cells harboring pETHrpA-C plasmid after 0, 1, 2, 3 or 4 h of IPTG induction (lanes 7 to 10, respectively). Lane 1: Molecular weight marker. **B** Recombinant HrpA-C in crude extract (lane 2),

S30 supernatant (lane 3) or S30 pellet (lane 4). Lane 1: Molecular weight marker. **C** Expression of recombinant HrpA middle region (HrpA-M) upon IPTG induction in *E. coli* BL21 (DE3). Crude protein extracts from: BL21 (DE3) cells after 0 or 4 h of IPTG induction (lanes 2 and 3, respectively); BL21 (DE3) cells harboring pETHrpA-M plasmid after 0, 1, 2, 3 or 4 h of IPTG induction (lanes 4 to 8, respectively). Lane 1: Molecular weight marker. **D** Recombinant HrpA-M in crude extract (lane 2), S30 supernatant (lane 3) or S30 pellet (lane 4). Lane 1: Molecular weight marker. **E-F** Expression of recombinant HrpA-M2 (**E**) and HrpA-M3 (**F**) upon IPTG induction in *E. coli* BL21 (DE3). Crude protein extracts from: BL21 (DE3) cells after 0, 1, 2, 3 or 4 h of IPTG induction (lanes 1, 2, 3, 4 and 5, respectively). M: Molecular weight marker.

A

```
   Waterman-Eggert score: 31;  14.5 bits; E(1) <  0.41
 18.5% identity (63.0% similar) in 27 aa overlap (44-70:34-60)

                50        60        70
   HrpA-M3 FYIQAINKEVKGKKPKGKEYLQAKLSA
           : ..  . . .: .   .:.:... .:
HHV-1 UL35 FIMDNNHPHPQGTQGAVREFLRGQAAA
                40        50        60

   Waterman-Eggert score: 28;  13.4 bits; E(1) <  0.66
 21.7% identity (56.5% similar) in 23 aa overlap (25-47:82-104)

               30        40
   HrpA-M3 SSPKSKLIPTLQEERDRLAFYIQ
           ..: . : :..  .:    : ..
HHV-1 UL35 DAPAAWLRPAFGLRRTYSPFVVR
                  90       100

   Waterman-Eggert score: 22;  11.3 bits; E(1) <  0.99
 30.8% identity (61.5% similar) in 13 aa overlap (69-81:10-22)

           70        80
   HrpA-M3 SAQNIDLISAQGI
           :. . : . : :.
HHV-1 UL35 STVTTDSVRALGM
          10        20
```

B

```
   Waterman-Eggert score: 44;  18.3 bits; E(1) <  0.14
 39.1% identity (65.2% similar) in 23 aa overlap (69-91:404-426)

           70        80        90
   HrpA-M3 SAQNIDLISAQGIEISGSDITAS
           .: :: :.:.  : :. .: . :
 HPV-16 L2 GAYNIPLVSGPDIPINITDQAPS
               410       420

   Waterman-Eggert score: 35;  15.4 bits; E(1) <  0.69
 25.0% identity (81.2% similar) in 16 aa overlap (35-50:455-470)

               40        50
   HrpA-M3 LQEERDRLAFYIQAIN
           :...: :: .... ..
 HPV-16 L2 LRKRRKRLPYFFSDVS
              460       470

   Waterman-Eggert score: 33;  14.7 bits; E(1) <  0.84
 21.1% identity (63.2% similar) in 19 aa overlap (53-71:216-234)

                 60        70
   HrpA-M3 VKGKKPKGKEYLQAKLSAQ
           . :..: ..  : .. . :
 HPV-16 L2 IPGSRPVARLGLYSRTTQQ
             220       230
```

C

```
   Waterman-Eggert score: 39;  16.9 bits; E(1) <  0.31
 21.2% identity (53.0% similar) in 66 aa overlap (27-87:187-252)

             30         40        50            60        70        80
   HrpA-M3 PKSKLIPT-LQEERDRLAFYIQAINKEVKGK----KPKGKEYLQAKLSAQNIDLISAQGIEISGSD
           :... .:  :.      ...: . ...: ::    : . . . . .: . :. .     . ::: :
     CD155 PNTSQVPGFLSGTVTVTSLWILVPSSQVDGKNVTCKVEHESFEKPQLLTVNLTVYYPPEVSISGYD
            190       200       210       220       230       240       250

   Waterman-Eggert score: 36;  15.9 bits; E(1) <  0.53
 31.8% identity (54.5% similar) in 22 aa overlap (32-53:37-58)

                  40        50
   HrpA-M3 IPTLQEERDRLAFYIQAINKEV
           .: .  .   :  :.:. : ::
     CD155 VPGFLGDSVTLPCYLQVPNMEV
             40        50

   Waterman-Eggert score: 34;  15.2 bits; E(1) <  0.7
 33.3% identity (72.2% similar) in 18 aa overlap (82-99:80-97)

                  90
   HrpA-M3 EISGSDITASKKLNLHAA
           . .: . . ::.:.. ::
     CD155 QTQGPSYSESKRLEFVAA
          80        90
```

Fig. S3 Regional homology between HrpA-M3 and viral proteins HHV-1 UL35 (**A**) and HPV-16 L2 (**B**) or cellular CD155 receptor of *Poliovirus* (**C**) determined by using the program LALIGN.

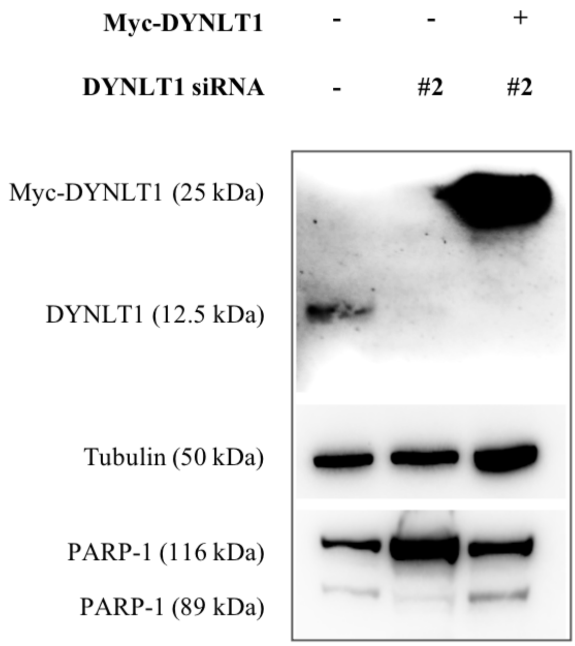

Fig. S4 HeLa cells were transfected with control RNA or with a specific siRNA for DYNLT1. After 24h of silencing, cells were transfected with a construct coding for Myc-tagged DYNLT1. Lysates were subjected to SDS-PAGE and analyzed by immunoblotting using specific antibody against PARP-1. Antibody against tubulin was used to verify loading while antibodies against DYNLT1 were used to check dynein silencing and over-expression.

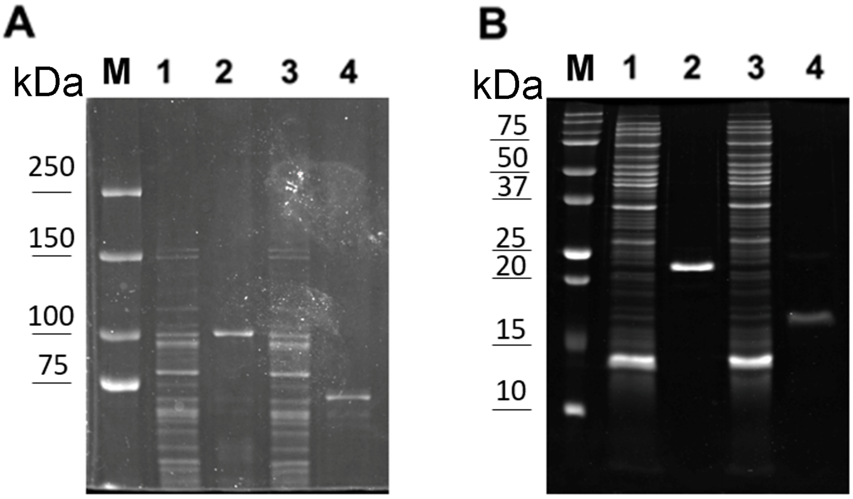

Fig.S5 A Coomassie Blue staining of proteins from cytosolic fraction of E. coli BL21 after incubation with Ni-NTA resin (lanes 1, 3) and purified HrpA-C and HrpA-M proteins (lanes 2, 4). **B** Coomassie Blue staining of proteins from cytosolic fraction of E. coli BL21 after incubation with Ni-NTA resin (lanes 1, 3) and purified HrpA-M2 and HrpA-M3 proteins (lanes 2, 4). M = molecular weight marker. The images were acquired using the ChemiDoc^{TM} MP Imaging System.

Tables

Table S1 Oligonucleotides used in this study.

Name	Nucleotide sequence (5' to 3')[a]
F_2HrpA	GGAGAACATATGAGCTTGCGTAACGGAGCCGTGGTGC
R_2HrpA	ATAGCAGGATCCTCTGAATCTGCTGCCTTTGGCAATACG
F_3HrpA	AATATTCATATGATTTCCGCACAAGGCATCGAAATCAGCG
R_3HrpA	AATTTTGGATCCTGCCCATTCTCCAGTACTGATAATGCC
F_{22}HrpA	CTCAACCATATGGATGCCTACGCACACCGTCATCTAAGCATT
F_{23}HrpA	AGCAATCATATGCCTACACAAAAAGCGGCTGAACTC
R_{21}HrpA	TGAGCGGGATCCGCTCGTGTTGTGCATTAAGATGCGT
R_{22}HrpA	TGAGTTGGATCCGGCTTCGATATTCAACTTGCCTTTGGT

[a]Restriction sites for cloning procedures are underlined.

Table S2 *In silico* analysis of the interaction between HrpA-M3 or UL35 and DYNLT1 (5jpw crystallographic or I-Tasser model): Free energy (KJ/mol).

	HrpA-M3	UL35
**DYNLT1 (5jpw crystallographic model)**	-34.98	-33.82
**DYNLT1 (I-Tasser model)**	-32.18	-39.70
